# RAF proteins exert both specific and compensatory functions during tumour progression of NRAS-driven melanoma

**DOI:** 10.1038/ncomms15262

**Published:** 2017-05-12

**Authors:** Coralie Dorard, Charlène Estrada, Céline Barbotin, Magalie Larcher, Alexandra Garancher, Jessy Leloup, Friedrich Beermann, Manuela Baccarini, Celio Pouponnot, Lionel Larue, Alain Eychène, Sabine Druillennec

**Affiliations:** 1 Institut Curie, Orsay F-91405, France; 2 INSERM U1021, Centre Universitaire, Orsay F-91405, France; 3 CNRS UMR 3347, Centre Universitaire, Orsay F-91405, France; 4 Université Paris Sud-11, Orsay F-91405, France; 5 Equipe Labellisée Ligue Nationale Contre le Cancer, Orsay F-91405, France; 6 Swiss Institute for Experimental Cancer Research (ISREC), Ecole Polytechnique Fédérale de Lausanne, Lausanne 1015, Switzerland; 7 Max F. Perutz Laboratories, Center for Molecular Biology, University of Vienna, Vienna 1030, Austria

## Abstract

NRAS and its effector BRAF are frequently mutated in melanoma. Paradoxically, CRAF but not BRAF was shown to be critical for various RAS-driven cancers, raising the question of the role of RAF proteins in NRAS-induced melanoma. Here, using conditional ablation of *Raf* genes in NRAS-induced mouse melanoma models, we investigate their contribution in tumour progression, from the onset of benign tumours to malignant tumour maintenance. We show that BRAF expression is required for ERK activation and nevi development, demonstrating a critical role in the early stages of NRAS-driven melanoma. After melanoma formation, single *Braf* or *Craf* ablation is not sufficient to block tumour growth, showing redundant functions for RAF kinases. Finally, proliferation of resistant cells emerging in the absence of BRAF and CRAF remains dependent on ARAF-mediated ERK activation. These results reveal specific and compensatory functions for BRAF and CRAF and highlight an addiction to RAF signalling in NRAS-driven melanoma.

## Abstract (web summary)

The melanoma-driver mutations in NRAS and BRAF are mutually exclusive but the contribution of RAF signalling downstream of NRAS remains to be clarified. Here, using mouse models, the authors show specific roles of each member of the RAF family at different stages of melanomagenesis.

Melanomagenesis is a multistep process arising from the transformation of skin melanocytes and leading to cutaneous melanoma, the most devastating type of skin cancer due to its highly metastatic potential^1^. Activation of the RAS/RAF/MEK/ERK signalling pathway is found in the vast majority of melanomas and often occurs through mutations of either NRAS or BRAF (15–20% and 40–50% cutaneous melanomas, respectively)^2^,^3^. These driver mutations represent a very early event found in benign melanocytic lesions, called nevi, and are maintained throughout all stages of invasive and metastatic melanoma. The development of drugs such as Vemurafenib and Dabrafenib, which selectively inhibit BRAF^V600E^, the most frequent BRAF mutation, represents a major breakthrough in melanoma treatment, triggering a response rate of 80% in phase I clinical trials and an overall survival benefit in phase III studies^4^,^5^. Unfortunately, resistances rapidly occur and most of responding patients relapse after 5–6 months of treatment^6^. In addition, such compounds cannot be used to treat half of melanoma patients, especially those harbouring NRAS mutations, since BRAF inhibitors paradoxically activate the MAPK/ERK pathway in BRAF wild-type cells^7^,^8^. Indeed, BRAF^V600E^ and NRAS mutations, although mutually exclusive, are not strictly equivalent and the contribution of RAF signalling downstream of NRAS remained to be clarified^9^,^10^,^11^.

Despite much attention being focused on BRAF mutant melanoma, NRAS was the first melanoma oncogene to be identified^12^, and mutations in NRAS, KRAS and HRAS are present in about 20, 2 and 1% of all melanomas, respectively^13^. NRAS^Q61K/R/L^ is the most frequent NRAS mutations, although point mutations at positions 12 and 13 also occur occasionally. Substantial evidence supports the RAF/MAPK pathway as a key downstream effector of oncogenic RAS in melanoma^14^, although other RAS downstream signalling pathways such as the phospho-inositide-3-kinase (PI3K), RAC1 and RAL also contribute to melanomagenesis^15^,^16^,^17^. Under physiological conditions in melanocytes, growth factors such as HGF and SCF activate RAS, which in turn binds its effectors, including RAF proteins^18^,^19^. In vertebrates, there exists three RAF proteins called ARAF, BRAF and CRAF that activate MAPK–ERK kinases (MEK1 and MEK2), which in turn activate extracellular signal-regulated kinases (ERK1 and ERK2)^20^. Using knockout (KO) mice, we have shown that BRAF and CRAF are not necessary for melanocyte lineage development, but are required for and play redundant functions in melanocyte stem cell self-maintenance^21^.

In NRAS-mutated human melanoma cell lines, CRAF has been proposed to be the major ERK activator^9^. However, paradoxical activation of the MAPK/ERK pathway by BRAF inhibitors is observed in NRAS-mutated melanoma and results from the recruitment of inhibited BRAF at the membrane where it acts as a scaffold to enhance CRAF activity^8^. Alternative mechanisms have been proposed that rely on either transactivation of RAF dimers or disruption of RAF inhibitory autophosphorylation by BRAF inhibitors^22^,^23^. A critical role for CRAF in RAS-driven skin tumorigenesis as well as in KRAS-induced lung cancer has been demonstrated in mouse models, suggesting that CRAF could be the prevalent effector in RAS-driven cancers^24^,^25^,^26^. Intriguingly, while BRAF is mutated in up to 50% of melanoma, no CRAF mutation was found so far, suggesting that oncogenic CRAF activation may not be sufficient for tumour initiation^27^. Therefore, the assumption that BRAF but not CRAF could be dispensable for NRAS-induced melanoma appears paradoxical.

To address this question, we re-evaluated the respective contribution of RAF kinases in RAS-induced melanoma using NRAS^Q61K^-induced mouse melanoma models in which single or compound ablation of *Braf* and *Craf* genes can be achieved in the melanocyte lineage upon tyrosinase promoter-driven Cre or Cre^ERT2^ expression^21^,^28^,^29^. Importantly, we previously reported that neither single nor double deletion of BRAF and CRAF affected the early development of the melanocyte lineage in a wild-type NRAS background. In addition, double but not single KO mice developed a hair-greying phenotype after the first hair molting, which occurs after 3 weeks of life^21^. Because the early development of the lineage is not altered in BRAF and CRAF KOs, these models allowed us to investigate the role of both RAF kinases in tumour progression from initiation to malignant melanoma.

Our results showed that BRAF, but not CRAF, was critical for the formation of early skin melanocytic lesions, including nevi-like benign tumours. Single KO of either CRAF or BRAF was not sufficient to prevent malignant tumour growth and maintenance *in vivo* and cell proliferation *in vitro*. In contrast, concomitant ablation of both BRAF and CRAF resulted in a complete blockage of tumour growth. This cooperative effect on cell proliferation was also observed in NRAS-mutated human melanoma cell lines. Finally, ablation of BRAF and CRAF leads to the emergence of resistant cells showing an ARAF-dependent reactivation of ERK and cell proliferation. Taken together, these results show that RAF signalling is absolutely required downstream of NRAS, and disclose both specific and compensatory functions for BRAF and CRAF kinases in NRAS-induced mouse melanoma.

## Results

### BRAF is required for early NRAS^Q61K^-driven melanomagenesis

The role of BRAF and CRAF kinases in NRAS-induced cutaneous melanoma was assessed by inactivating *Raf* genes in the *Tyr::NRAS*^Q61K^/^o^*;Ink4a*^+/−^ transgenic mouse model^28^. Constitutive expression of an activated form of human NRAS (NRAS^Q61K^) in the melanocytic lineage results in spontaneous cutaneous melanoma development, a process that is accelerated on an *Ink4a*-deficient background. Inactivation of *Raf* genes into the melanocyte lineage was performed by crossing double *Braf*^f/f^;*Craf*^f/f^ or single *Braf*^f/f^;*Craf*^+/+^ or *Braf*^+/+^;*Craf*^f/f^ conditional KO mice^21^ to the *Tyr::Cre/*^o^ transgenic mice, in which the *Tyrosinase* promoter is active from embryonic day E10.5 to adulthood^30^. Conversion of floxed alleles by Cre recombinase and loss of BRAF and/or CRAF expression were demonstrated previously^21^. The resulting genotype of mouse lines is (*Braf*^f/f^;*Craf*^+/+^;*Tyr::NRAS*^Q61K^/^o^*;Ink4a*^+/−^;*Tyr::Cre/*^o^), (*Braf*^+/+^; *Craf*^f/f^;*Tyr::NRAS*^Q61K^/^o^*;Ink4a*^+/−^;*Tyr::Cre/*^o^) and (*Braf*^f/f^;*Craf*^f/f^;*Tyr::NRAS*^Q61K^/^o^*;Ink4a*^+/−^;*Tyr::Cre/*^o^), respectively, named BRAF KO, CRAF KO and BRAF/CRAF KO in the manuscript. Mice were also crossed to *Dct::LacZ/*^o^ reporter mice in order to trace cells from the melanocyte lineage^31^.

**Figure 1:**
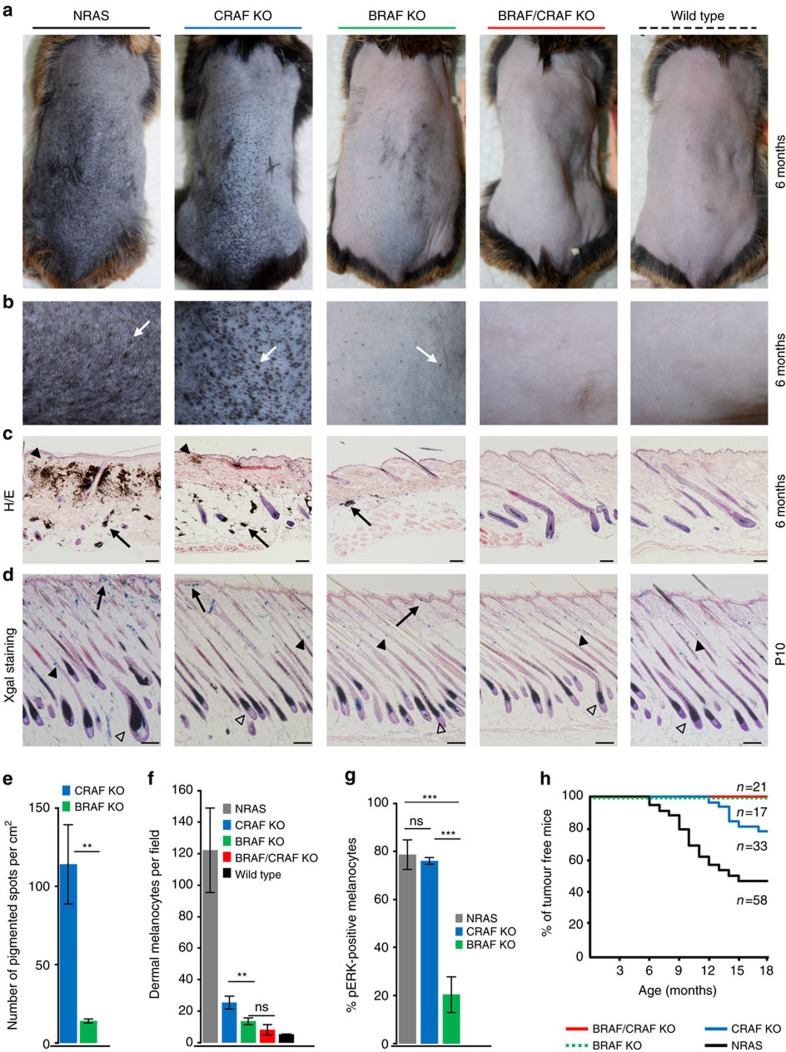
Effect of early deletion of BRAF and/or CRAF on hyperpigmentation and melanoma formation induced by NRAS^Q61K^. (**a**) Representative pictures of depilated back skin from 6-month-old mice with the indicated genotypes compared to control NRAS (*Tyr::NRAS*^Q61K^/^o^*;Ink4a*^+/−^) and wild-type mice showing the effect of CRAF, BRAF or BRAF/CRAF loss on hyperpigmented phenotype. (**b**) High-magnification pictures of bottom part of depilated back skin. Skin lesions are indicated by white arrows. (**c**) Haematoxylin and eosin (H&E) staining of representative back skin histological sections from 6-month-old control, mutant and wild-type mice. Typical melanin deposits and melanocyte clusters are shown in the papillary dermis (black arrowheads) and in the subcutaneous fat layer (arrows). Scale bar, 100 μm. (**d**) X-gal staining of representative skin sections from 10-day-old mice. Melanocytes are observed in papillary dermis (arrow) and in the shaft and the bulb of the hair follicule (black and open arrowheads, respectively). Scale bar, 100 μm. (**e**) Number of pigmented spots per cm^2^ on bottom back skin of 6-month-old mice (**b**). (**f**) Number of dermal melanocytes per microscopic field (× 10 objective) in skin sections from 10-day-old mice (**d**). (**g**) Quantification of pERK-positive melanocytes in the skin from RAS control (*Tyr::NRAS*^Q61K^/^o^*; Ink4a*^+/−^; *Tyr::Cre/*^o^*; Dct::LacZ*/^o^), CRAF KO (*Braf*^+/+^; *Craf*^f/f^; *Tyr::NRAS*^Q61K^/^o^*; Ink4a*^+/−^; *Tyr::Cre/*^o^*; Dct::LacZ*/^o^) or BRAF KO (*Braf*^f/f^; *Craf*^+/+^; *Tyr::NRAS*^Q61K^/^o^*; Ink4a*^+/−^; *Tyr::Cre/*^o^*; Dct::LacZ*/^o^) mice at P10. (**h**) Kaplan–Meier curves of melanoma incidence. In all, 21% of CRAF-deficient mice (7 of 33) develop melanoma with a latency of 15.7±1.8 months compared to 53.5% of control mice (31 of 58) in 10.8±2.7 months. No melanoma was observed in BRAF-deficient or BRAF/CRAF-deficient mice (17 and 21 mice, respectively, per cohort). **P value<0.01, ***P value<0.001 compared by Student's *t*-test. ns, not significant. All data are represented as mean±s.d. Overall, 123 males on a SV129/C57Bl6 mixed genetic background were used for **a**–**c**,**e**,**h** at the indicated age. Forty males and females on a SV129/C57Bl6 mixed genetic background were used for **d**,**f**,**g** at the p10.

In agreement with previous reports^28^,^32^,^33^, *Tyr::NRAS*^Q61K^/^o^*;Ink4a*^+/−^ control mice, when compared to wild-type mice, rapidly started to develop a highly hyperpigmented phenotype within the first week, which was remarkable on dorsal skin after depilation at 6 months (Fig. 1a). This phenotype was associated with the presence of a high number of black dome-shaped nevi (Fig. 1b). As shown on histological sections of adult back skin (Fig. 1c), hyperpigmentation was linked to an abnormal localization of melanin deposits and numerous melanocytes in the papillary dermis close to the epidermis, compared to wild-type mice. Melanin-containing melanophages are also present particularly in the subcutaneous fat layer. Importantly, staining for β-galactosidase activity on dorsal skin from newborn (P0.5) animals indicated that NRAS^Q61K^ did not affect the normal development of the melanocytic lineage before birth (Supplementary Fig. 1), as previously reported^33^. In contrast, at P10, while melanocytes were mainly located in the hair shaft and the bulb in normal mice, they accumulated in clusters at the epidermis/dermis junction and in intrafollicular part of the dermis in NRAS^Q61K^ control mice (Fig. 1d) leading to hyperpigmentation and nevi emergence^33^.

Deletion of both BRAF and CRAF upon Cre expression in melanocytic cells induced a complete reversion of the hyperpigmented phenotype since double KO mice were indistinguishable from wild-type mice with a normal light skin (Fig. 1a). Like in wild-type mice, only a very few number of weakly pigmented and flat spots were present (Fig. 1b). No abnormal localization of melanocytes or melanin deposits was observed in adult skin (Fig. 1c). Likewise, LacZ-positive cells at P10 were mainly located in the hair follicle as found in wild-type animals (Fig. 1d). Of note, the development of the normal melanocytic lineage at stages preceding the appearance of the NRAS phenotype was not affected in BRAF/CRAF KO animals (Supplementary Fig. 1), as previously reported^21^. These observations demonstrated that RAF signalling is required downstream of NRAS^Q61K^ for the development of the associated phenotype.

Single deletion of either BRAF or CRAF gave rise to markedly different phenotypes (Fig. 1a–c). While CRAF-deficient mice developed only a weakly altered hyperpigmented phenotype, BRAF-deficient mice displayed a nearly complete reversion of the phenotype. In CRAF-deficient mice, dorsal skin appeared dark, with numerous nevi. Histological analyses revealed the presence of aberrant location of melanocytes and melanin deposit in the dermis and fat layer. This suggested that CRAF was weakly required for NRAS-induced hyperpigmentation. In contrast, BRAF deletion resulted in a pigmentation pattern similar to that of double KO or wild-type mice. Only a few numbers of pigmented spots were observed, and skin sections were nearly unpigmented. We quantified the skin lesions by counting the number of pigmented spots per cm^2^ on the back of BRAF KO or CRAF KO mice (Fig. 1e). Tenfold more spots were counted in CRAF-deficient compared to BRAF-deficient skin portions. In addition, these skin lesions displayed characteristics of highly pigmented and dome-shaped nevi in CRAF KO animals, whereas they were very weakly pigmented and remained flat in BRAF KO animals. We analysed the distribution of melanocytes in P10 skins of both single KOs and counted the number of LacZ-positive cells per microscopic field in the dermis (Fig. 1f). The localization of melanocytes in BRAF-deficient mice was not different from that of double KO animals. In contrast, we observed a significantly higher number of dermal melanocytes in CRAF KO compared to BRAF KO, in agreement with the higher number of nevi found in adult skins. Taken together, these results show that, although BRAF is dispensable for normal melanocyte lineage development and homeostasis^21^, it is critical for NRAS-induced hyperpigmentation and nevi formation. At the molecular level, we looked at NRAS-induced ERK activation at P10 in the melanocytic lineage of control, BRAF KO and CRAF KO mice by immunohistochemistry. As shown in Fig. 1g and Supplementary Fig. 2, phospho-ERK staining was highly compromised in BRAF-deficient but not in CRAF-deficient melanocytes in comparison to control melanocytes from NRAS^Q61K^ mice at P10, thus strengthening the notion that CRAF alone is not able to compensate for BRAF absence during early stages of melanomagenesis.

We then looked at the emergence of melanoma in single and double *Raf* KO animals. In all, 53.5% of *Tyr::NRAS*^Q61K^/^o^*;Ink4a*^+/−^ control mice (31 out of 58) developed melanoma with an average latency of 10.8±2.7 months (Fig. 1h). No tumour was observed in BRAF KO and BRAF/CRAF KO mice, in agreement with the lack of hyperpigmented phenotype upon early BRAF deletion, thereby confirming the requirement of BRAF for tumour initiation. Conversely, 21% of CRAF KO animals (7 out of 33) displayed melanoma with an average higher latency of 15.7±1.8 months. The lower incidence and longer latency compared to control mice could be either in favour of a role for CRAF in tumour growth or due to the mild effect of its early deletion on hyperpigmentation and nevi appearance as described above.

Taken together, these results demonstrate a specific function for BRAF during the early stages of melanomagenesis that cannot be compensated by CRAF.

### BRAF and CRAF compensation in late melanomagenesis

In order to study the respective roles of BRAF and CRAF in melanoma growth after tumour initiation, we generated a second NRAS-mutated melanoma model in which *Raf* gene ablation is controlled in a spatiotemporal manner by using the *Tyr::CreERT2*^/o^ transgene expressing a tamoxifen-inducible Cre recombinase^29^. Control animals (67%, 117 of 174) developed cutaneous melanoma within ∼8 months after birth (8.1±2.3 months).

**Figure 2:**
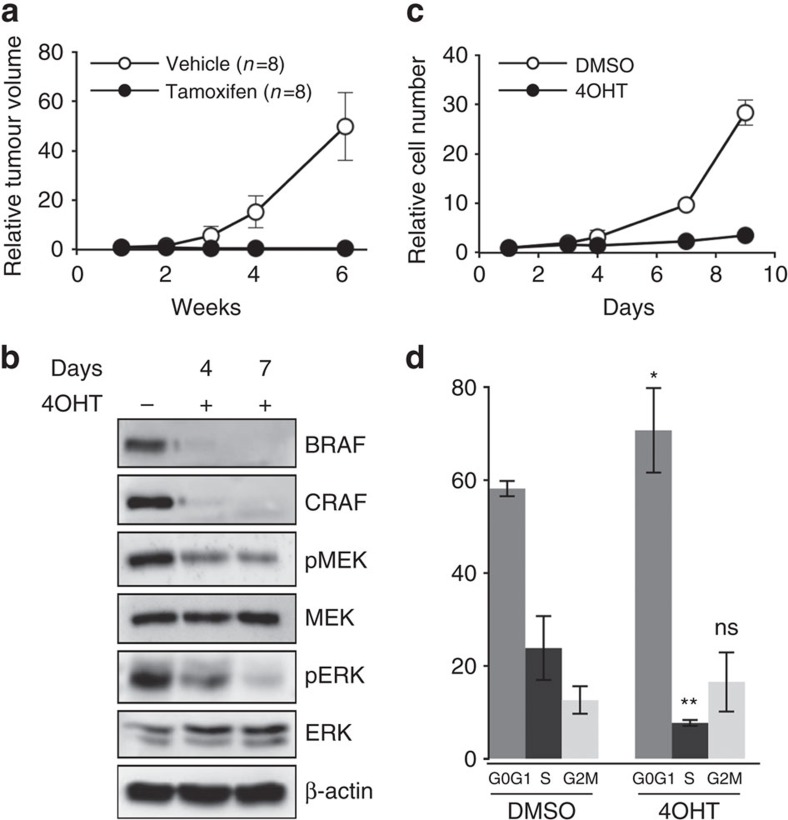
RAF signalling is required for cell proliferation and tumour growth in NRAS^Q61K^-induced murine melanoma. (**a**) A melanoma from an untreated *Braf*^f/f^*;Craf*^f/f^*;Tyr::NRAS*^Q61K^/^o^*;Ink4a*^+/−^*;Tyr::CreERT2*/^o^ mouse was cut into small pieces and subcutaneously grafted into two groups of nude mice that were treated either with tamoxifen or vehicle for 2 weeks. The effect on tumour growth was assessed by measuring tumour volume over a 6-week period. Tumour volumes are plotted relative to the initial volume at the start of treatment. This experiment is representative of three independent experiments requiring 48 Swiss Nu/Nu females (6-week-old) for one primary tumour from a 1-year-old female on a SV129/C57Bl6 mixed genetic background. (**b**) Western blot analysis of BRAF and CRAF protein levels and MEK and ERK activation levels (pMEK and pERK, respectively) in protein lysates from culture in **c** on days 4 and 7 of 4OHT treatment compared to DMSO-treated culture. Total MEK, total ERK and β-actin are shown as a loading control. (**c**) Growth curve analysis of melanoma cell culture established from an untreated *Braf*^f/f^;*Craf*^f/f^*;Tyr::NRAS*^Q61K^/^o^*;Ink4a*^+/−^*;Tyr::CreERT2*/^o^ primary mouse tumour in response to 4OHT or DMSO for 9 days. Cell number is plotted relative to the initial number of cells at the start of treatment. Data are representative of three independent experiments. (**d**) Cell cycle analysis by FACS from culture in **c** on day 6 of 4OHT treatment compared to DMSO-treated culture. Data are the mean value of three independent experiments. *P value <0.05 and **P value <0.01 compared by Student's *t*-test. ns, not significant. All data are represented as mean±s.d.

We first attempted to look at the effect of *Raf* gene ablation by directly treating the tumours of the different genotypes with tamoxifen but did not observe tumour regression whatever the genotype studied; but in most cases complete recombination of floxed *Raf* alleles was not achieved (Supplementary Fig. 3). Therefore, we looked at the consequence of *Raf* genes ablation on tumour growth by comparing the effect of tamoxifen and vehicle treatment on the same primary tumour subcutaneously transplanted into two identical cohorts of nude mice. In parallel, we also looked at the effect of *Raf* genes ablation *in vitro* on melanoma cultures established from primary mouse tumours and treated with 4-hydroxy-tamoxifen (4OHT). treatment of control tumours with tamoxifen or control cultures with 4OHT did not affect tumour growth and cell proliferation, respectively (Supplementary Fig. 4). As shown on Fig. 2a, tamoxifen administration totally blocked tumour growth in mice grafted with double *Braf*^f/f^;*Craf*^f/f^ tumours, compared to vehicle. Consequently, we were unable to collect any tumour material at the end of the experiment in tamoxifen-treated animals. *In vitro*, 4OHT-induced recombination was highly efficient in double *Braf*^f/f^;*Craf*^f/f^ melanoma cell culture since BRAF and CRAF expression was nearly undetectable on day 4 and completely abolished on day 7 of treatment (Fig. 2b). Moreover, the concomitant loss of BRAF and CRAF impaired MAPK pathway activation as evidenced by western blot analysis of MEK and ERK phosphorylation status (Fig. 2b) and strongly inhibited cell proliferation (Fig. 2c). Cell cycle analyses revealed that BRAF and CRAF double KO cells accumulated into G0/G1 phase resulting in a fewer proportion of cells in S-phase compared to dimethylsulphoxide (DMSO)-treated samples (Fig. 2d). Taken together, these results demonstrated that the RAF/MAPK pathway is absolutely required for NRAS^Q61K^-driven tumour growth in melanoma.

**Figure 3:**
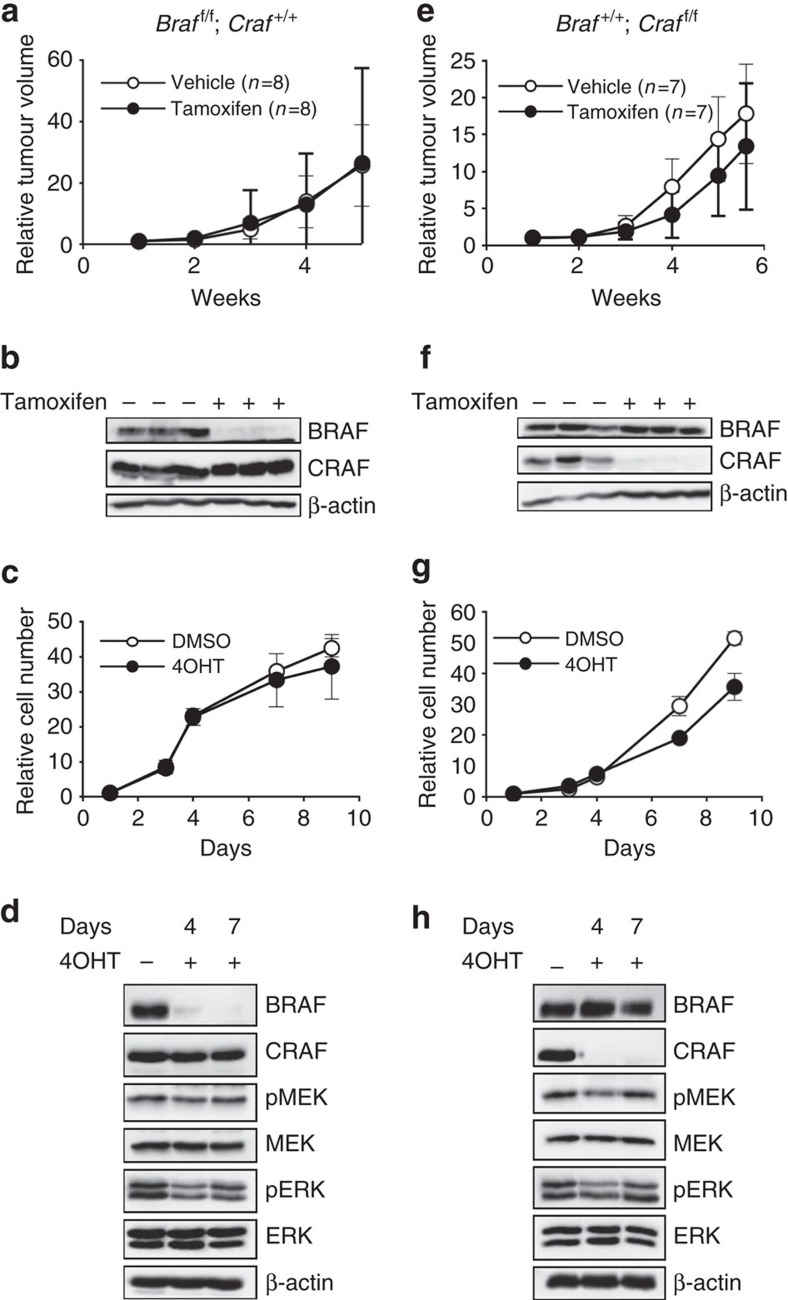
Compensatory functions of BRAF and CRAF for cell proliferation and tumour growth in NRAS^Q61K^-induced murine melanoma. (**a**,**e**) A melanoma from an untreated *Braf*^f/f^*;Craf*^+/+^*;Tyr::NRAS*^Q61K^/^o^*;Ink4a*^+/−^*;Tyr::CreERT2*/^o^ or *Braf*^+/+^*;Craf*^f/f^*;Tyr::NRAS*^Q61K^/^o^*;Ink4a*^+/−^*;Tyr::CreERT2*/^o^ mouse (**a**,**e**, respectively) was cut into small pieces and subcutaneously grafted into two groups of nude mice and experimented as in Fig. 2a. These experiments required 48 Swiss Nu/Nu females (6-week-old) for each primary tumour from a 5-month-old female and a 1-year-old male on a SV129/C57Bl6 mixed genetic background, respectively. (**b**,**f**) Western blot analysis for BRAF and CRAF expression at the end of treatment by tamoxifen or vehicle in three individual and representative tumours from **a**,**e**, respectively. β-actin is used as a loading control. (**c**,**g**) Growth curve analysis of melanoma cell culture established from an untreated *Braf*^f/f^*;Craf*^+/+^*;Tyr::NRAS*^Q61K^/^o^*;Ink4a*^+/−^*;Tyr::CreERT2*/^o^ or *Braf*^+/+^*;Craf*^f/f^*;Tyr::NRAS*^Q61K^/^o^*;Ink4a*^+/−^*;Tyr::CreERT2*/^o^ primary mouse tumour (**c**,**g**, respectively) in response to 4OHT or DMSO for 9 days as in Fig. 2c. (**d**,**h**) Western blot analysis of BRAF and CRAF protein levels and MEK and ERK activation levels in protein lysates from culture in **c**,**g** respectively, as in Fig. 2b. All data are represented as mean±s.d.

The same strategy was used to study *in vivo* and *in vitro* the respective functions of BRAF or CRAF in tumours from single *Braf*^f/f^;*Craf*^+/+^ and *Braf*^+/+^;*Craf*^f/f^ KOs. Western blot analyses confirmed the efficient recombination of floxed alleles upon Cre activation by showing a complete loss of BRAF or CRAF expression in solid tumours and in cultures after treatment by tamoxifen or 4OHT, respectively (Fig. 3b,d and Fig. 3f,h respectively). Interestingly, BRAF loss had no effect on either tumour growth or cell proliferation (Fig. 3a,c). CRAF depletion reproducibly induced a very weak effect on tumour growth and a mild slowdown of cell proliferation (Fig. 3e,g). Following BRAF or CRAF loss *in vitro*, only a modest decrease in MEK and ERK phosphorylation was observed on day 4 of treatment and the levels of MEK and ERK activation returned to normal on day 7. These results demonstrated that, in contrast to the striking effect obtained with the double BRAF/CRAF deletion, neither CRAF nor BRAF loss alone was sufficient to efficiently block tumour growth and cell proliferation. This strongly suggested that, while CRAF was not able to compensate for BRAF activity during early stages of melanomagenesis (Fig. 1), compensatory effects between BRAF and CRAF could rapidly take place in melanoma cells.

**Figure 4:**
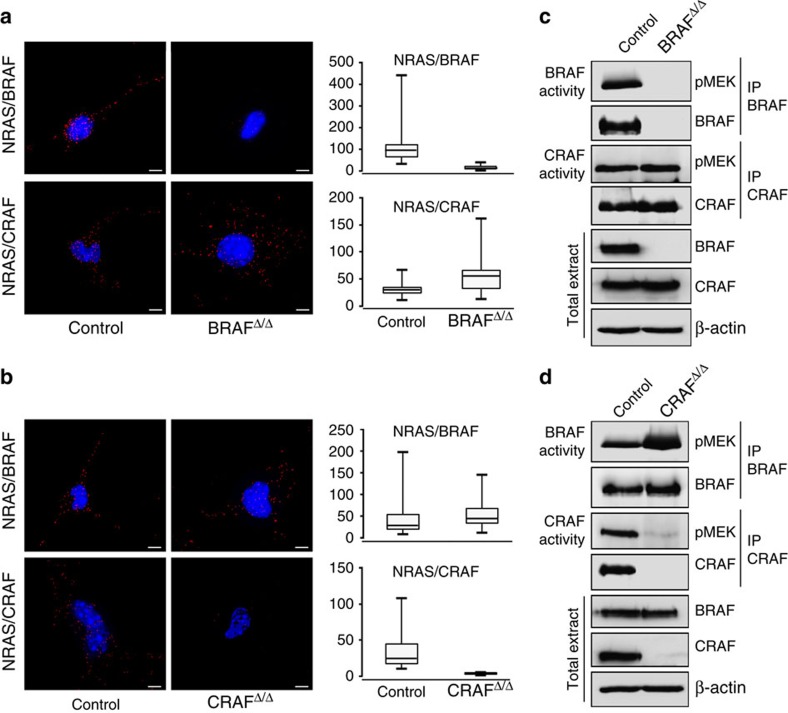
NRAS-binding and kinase activity of BRAF and CRAF in NRAS-induced melanoma. (**a**,**b**) PLA showing NRAS/BRAF and NRAS/CRAF complexes in BRAF^Δ/Δ^ and CRAF^Δ/Δ^ murine melanoma cultures compared to the parental cultures (**a**,**b**, respectively). NRAS/BRAF and NRAS/CRAF interactions in cultures established from Fig. 3c,g were visualized as red dots by using a fluorescent microscope. Cell nuclei were stained with 4,6-diamidino-2-phenylindole (DAPI). Box plots represent the average number of dots per cell. Lower, median and upper quartiles are shown, with whiskers extending to the lowest and highest values. Scale bar, 100 μm. (**c**,**d**) BRAF and CRAF *in vitro* kinase assays in BRAF^Δ/Δ^ or CRAF^Δ/Δ^ cultures compared to parental control cultures (**c**,**d**, respectively). BRAF or CRAF were immunoprecipitated and their intrinsic kinase activity against kinase-inactive MEK was determined by western blotting using anti-pMEK antibody. Immune complexes and total extracts were immunoblotted with anti-BRAF and anti-CRAF antibodies. β-actin was used as a loading control. BRAF^Δ/Δ^ and CRAF^Δ/Δ^ refer to *Braf*^*Δ/Δ*^*;Craf*^+/+^*;Tyr::NRAS*^Q61K^/^o^*;Ink4a*^+/−^*;Tyr::CreERT2*/^o^ *and Braf*^+/+^*;Craf*^*Δ/Δ*^*;Tyr::NRAS*^Q61K^/^o^*; Ink4a*^+/−^*; Tyr::CreERT2*/^o^, respectively and control to the corresponding parental cultures.

To investigate the molecular mechanisms underlying this differential requirement of RAF kinases at different stages of melanomagenesis, we used *in situ* proximity ligation assays (PLA) to look at the ability of endogenous BRAF and CRAF to interact with NRAS first in cells from full-blown melanoma. Results in Fig. 4a,b showed the presence of both NRAS/BRAF and NRAS/CRAF complexes in control parental cells. The specificity of the PLA signal was confirmed by the absence of signal in BRAF- or CRAF-depleted cells. These findings were confirmed by co-immunoprecipitation experiments using an NRAS-specific antibody that enabled to detect interactions between NRAS and both BRAF and CRAF (Supplementary Fig. 5). In agreement with the lack of effect of BRAF depletion on growth curve and cell proliferation, the kinase activity of CRAF was comparable in both parental and BRAF KO cells (Fig. 4c). In contrast, BRAF kinase activity was increased in CRAF KO cells (Fig. 4d), explaining the compensatory effect of BRAF in the absence of CRAF. Taken together, these results led us to propose that, while CRAF acts as the predominant kinase downstream of NRAS^Q61K^ to sustain melanoma growth, its loss can be rapidly compensated by BRAF. They also showed that either BRAF or CRAF alone could trigger the level of ERK activation required for tumour maintenance. In contrast, only BRAF was able to sustain the level of ERK activation required to initiate melanomagenesis (Fig. 1g). Therefore, we looked at the ability of BRAF and CRAF to bind NRAS early during melanomagenesis. *Tyr::NRAS*^Q61K^/^o^*;Ink4a*^+/−^;*Tyr::Cre/*^o^ mice were crossed with the *mT/mG* reporter mouse that expresses membrane-targeted green fluorescent protein (GFP) after Cre-mediated excision^34^. Fluorescence-activated cell sorting (FACS)-sorted GFP-positive melanocytes from the skin of 10-day-old animals were then submitted to PLA assay (Supplementary Fig. 6). In contrast with what is observed in established control melanoma cells (Supplementary Fig. 6b,d), NRAS/CRAF complexes were strikingly much less abundant than NRAS/BRAF complexes in melanocytes at P10 (Supplementary Fig. 6a,c), thus explaining why CRAF can compensate for BRAF absence in melanoma cells but not in early melanocytic lesions.

### BRAF and CRAF are both required for NRAS human melanoma

**Figure 5:**
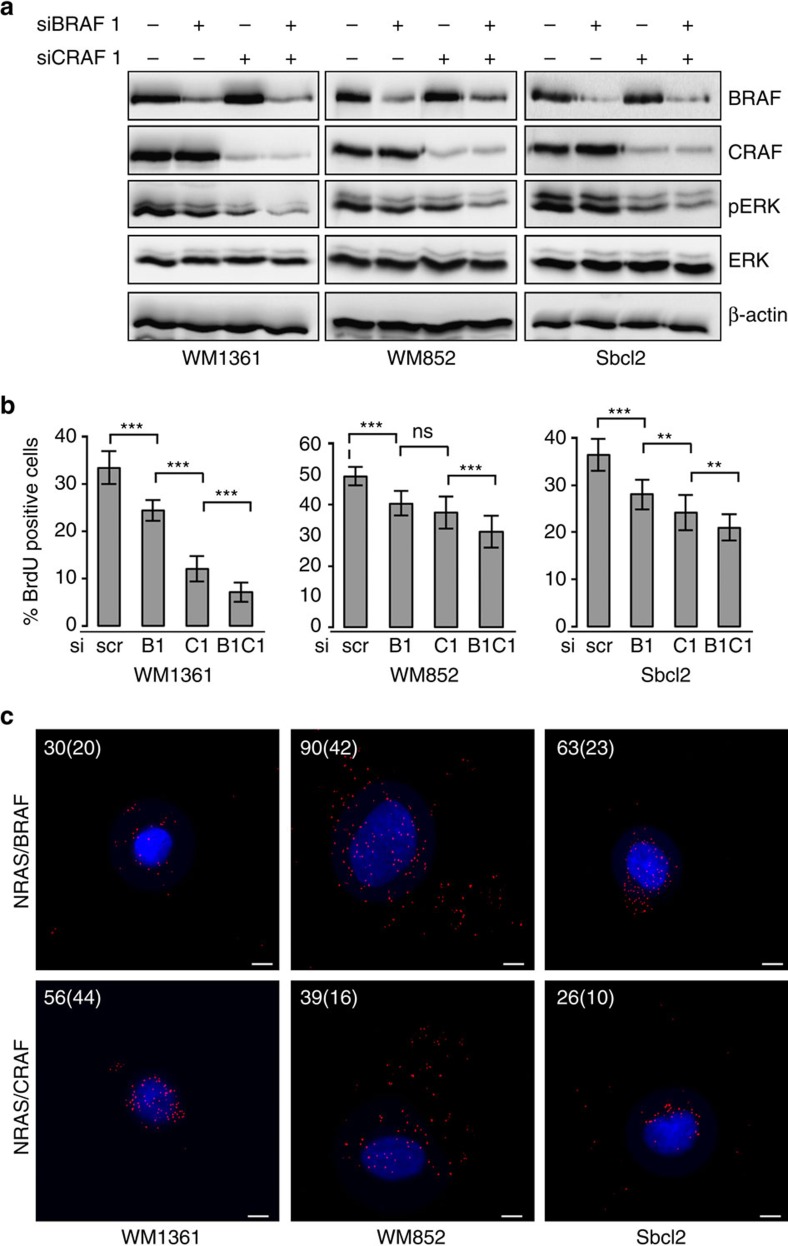
NRAS-mutated human melanoma cells require both BRAF and CRAF for ERK activation and proliferation. (**a**) Western blot analysis of BRAF and CRAF protein expression and pERK activation in NRAS-mutated human melanoma cell lines (WM1361, WM852 and Sbcl2) transfected with the scrambled control (−) or short interfering RNA to BRAF and/or CRAF (siBRAF1 and siCRAF1). Total ERK and β-actin are used as a loading control. (**b**) Proliferation rate in WM1361, WM852 and Sbcl2 cells transfected with scrambled control (scr), BRAF siRNA (B1), CRAF siRNA (C1) or BRAFsiRNA/CRAFsiRNA (B1C1) was measured after BrdU incorporation during 3 hours. **P-value<0.01 and ***P-value<0.001 compared by Student's *t*-test. All data are represented as mean±s.d. (**c**) PLA showing NRAS/BRAF and NRAS/CRAF complexes in WM1361, WM852 and Sbcl2 cells. Numbers in white indicate the average number of dots per cell. Numbers between brackets represent s.d.'s. Scale bar, 100 μm.

It has been previously reported that in RAS-mutated human melanoma cell lines, CRAF but not BRAF was required for MAPK activation presumably because BRAF cannot be activated when RAS is mutated^9^. Our observations in the mouse model being in apparent discrepancy with these conclusions, we re-investigated the respective functions of BRAF and CRAF by using RNA interference experiments in three human melanoma cell lines harbouring NRAS mutations: WM1361 (NRAS^Q61K^), WM852 (NRAS^Q61R^) and Sbcl2 (NRAS^Q61K^). Western blot analyses showed the efficiency of BRAF and/or CRAF depletion by two distinct short interfering RNA (siRNA) couples (siBRAF1/siCRAF1 and siBRAF2/siCRAF2 in Fig. 5a and Supplementary Fig. 7a, respectively). Proliferation rate was also measured after 5-bromodeoxyuridine (BrdU) incorporation (Fig. 5b and Supplementary Fig. 7b). Consistent results were obtained in the three different cell lines. Downregulation of CRAF expression induced a decrease in ERK activation as reported^9^ and affected cell proliferation. However, BRAF depletion also affected ERK phosphorylation and proliferation, although the effects were less pronounced than upon CRAF depletion. More importantly, the concomitant downregulation of both BRAF and CRAF expression strongly inhibited ERK phosphorylation and proliferation. Taken together, these results indicated that when CRAF is knocked down in NRAS-mutated human melanoma cell lines, BRAF is able to compensate to maintain MAPK activation and proliferation. Accordingly, PLA experiments proved that not only CRAF but also BRAF could form complexes with NRAS in NRAS-mutated human melanoma cell lines (Fig. 5c). Therefore, the compensatory effects of RAF kinases is a conserved mechanism between human and murine NRAS-driven melanoma, since neither CRAF, nor BRAF loss alone is sufficient to block melanoma cell proliferation in both species.

### ARAF compensates for BRAF and CRAF absence

**Figure 6:**
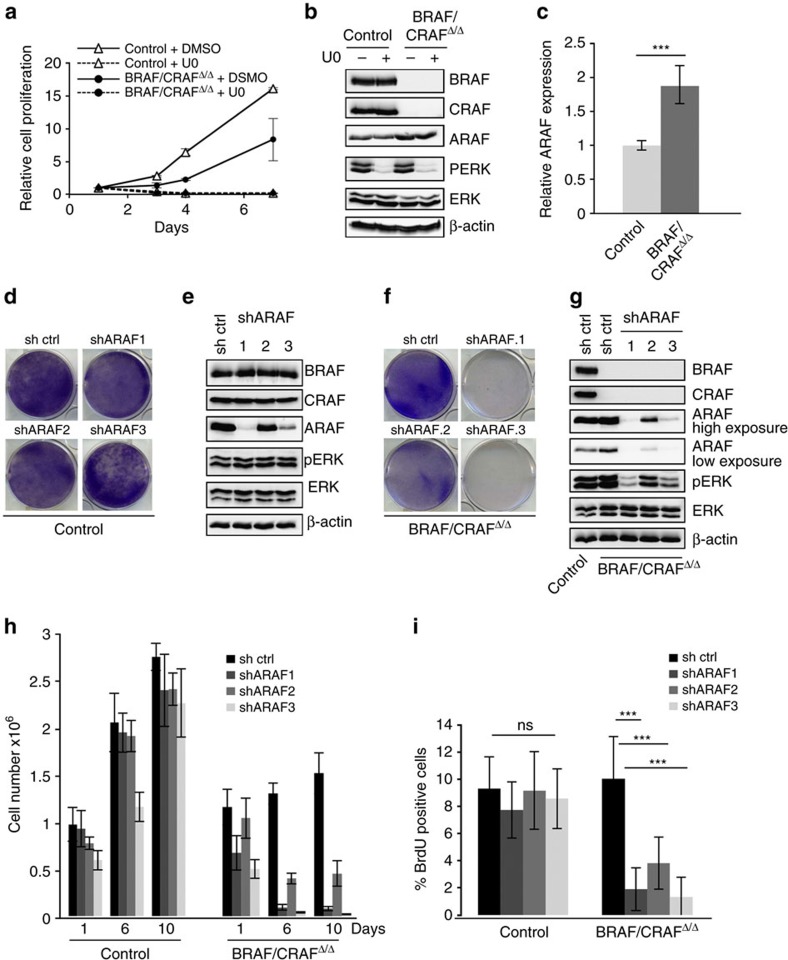
ARAF is required for the survival NRAS^Q61K^-induced murine melanoma cell lines in absence of BRAF and CRAF. (**a**) Growth curve analysis of resistant double knockout murine melanoma cell culture BRAF/CRAF^Δ/Δ^ (circles) compared to its parental control culture (triangles) for 6 days in presence of 10 μM U0126 (U0) or DMSO. Cell number is plotted relative to the initial number of cells at the start of treatment. Data are representative of three independent experiments. (**b**) Western blot analysis of ARAF, BRAF and CRAF protein expression and pERK activation in BRAF/CRAF^Δ/Δ^ and parental control cultures after treatment by 10 μM U0 or DMSO. Total ERK and β-actin are used as a loading control. (**c**) qRT–PCR analysis of ARAF expression in BRAF/CRAF^Δ/Δ^ and parental control cultures. ***P value<0.001 compared by Student's *t*-test. (**d**,**f**) Parental control and BRAF/CRAF^Δ/Δ^ cultures (**d**,**f**, respectively) were infected by lentiviruses encoding control shRNA or targeting ARAF (shARAF.1; shARAF.2; shARAF.3). After puromycin selection, cells were stained with crystal violet. (**e**,**g**) Western blot analysis of ARAF, BRAF and CRAF protein expression levels and pERK activation in parental control and BRAF/CRAF^Δ/Δ^ cultures (**e**,**g**, respectively) after infection by control or ARAF shRNA encoding viruses and selection. Total ERK and β-actin are used as a loading control. Control refers to *Braf*^f/f^*;Craf*^f/f^*;Tyr::NRAS*^Q61K^/^o^*;Ink4a*^+/−^*;Tyr::CreERT2*/^o^ parental culture and BRAF/CRAF^Δ/Δ^ refers to *Braf*^*Δ/Δ*^*;Craf*^*Δ/Δ*^*;Tyr::NRAS*^Q61K^/^o^*;Ink4a*^+/−^*;Tyr::CreERT2*/^o^ double knockout culture. (**h**) Cell counting of parental control and BRAF/CRAF^Δ/Δ^ cultures infected with lentiviruses encoding control or ARAF shRNA on days 1, 6 and 10 after puromycin selection. (**i**) Proliferation rate in parental control and BRAF/CRAF^Δ/Δ^ cultures infected by lentiviruses encoding control or ARAF shRNA was measured after BrdU incorporation during 9 h. ***P value<0.001 compared by Student's *t*-test. ns, not significant. All data are represented as mean±s.d.

As shown in Fig. 2c, 4OHT-induced concomitant loss of BRAF and CRAF completely inhibited cell proliferation. However, at the end of the treatment, we observed the emergence of resistant clones that enabled us to establish double BRAF and CRAF KO melanoma cultures. These resistant cultures were not due to an escape from the treatment since the total absence of BRAF and CRAF protein expression was stably maintained during passages (Fig. 6b). Western blot analyses revealed comparable levels of ERK phosphorylation between resistant cells and control parental cells (Fig. 6b), demonstrating that the MAPK pathway, which was initially inhibited upon treatment of parental cells with 4OHT (Fig. 2b), was reactivated in resistant cells. Treatment by U0126 MEK inhibitor (U0) strongly inhibited ERK phosphorylation and cell proliferation of both control and resistant cells (Fig. 6a,b), indicating that the implemented mechanism of resistance was still dependent of the RAF/MEK/ERK pathway. In addition, western blot and quantitative reverse transcriptase PCT (qRT–PCR) analyses (Fig. 6b,c) demonstrated that ARAF expression, the third member of the RAF kinases family, was increased in double BRAF- and CRAF-deficient resistant cultures. We therefore examined whether ARAF could be involved in this resistance by using short hairpin RNA (shRNA) vectors targeting ARAF. We tested three different sequences and identified two shRNAs (shARAF.1 and shARAF.3) that efficiently downregulated ARAF expression in control and resistant cells (Fig. 6e,g, respectively). ARAF downregulation induced a decrease in ERK phosphorylation in absence of BRAF and CRAF, whereas its depletion had no effect on MAPK activation in control cells. Interestingly, the levels of ARAF expression achieved by the different shRNAs strictly correlated with the levels of ERK phosphorylation. Crystal violet staining of mass cultures at the end of puromycin selection (Fig. 6d,f) as well as growth curve analysis (Fig. 6h) and BrdU incorporation experiments (Fig. 6i) revealed that ARAF downregulation prevented cell proliferation in resistant cells but not in control parental cells expressing BRAF and CRAF. Taken together, these data strongly supported that loss of BRAF and CRAF in NRAS-mutated murine melanoma cells induced resistances involving a compensatory effect of ARAF.

**Figure 7:**
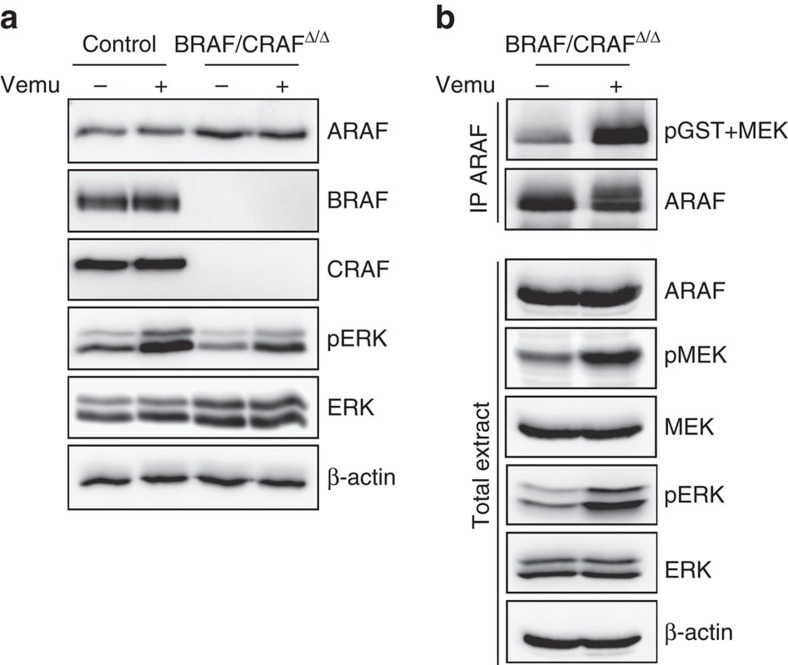
Vemurafenib induces ERK paradoxical activation in BRAF- and CRAF-decifient NRAS-induced melanoma by increasing ARAF kinase activity. (**a**) Western blot analysis of ERK activation (pERK) and ARAF, BRAF and CRAF protein expression in parental control and BRAF/CRAF^Δ/Δ^ cultures after treatment with 1 μM Vemurafenib (Vemu) or DMSO during 1 h. Total ERK and β-actin are used as loading controls. (**b**) ARAF *in vitro* kinase assays in BRAF/CRAF^Δ/Δ^ cultures after treatment with 1 μM Vemurafenib or DMSO during 1 h. ARAF was immunoprecipitated and its intrinsic kinase activity was measured on kinase-inactive MEK as substrate by western blotting using anti-pMEK antibody. Immune complexes and total cell extracts were immunoblotted with anti-ARAF, pMEK, MEK, pERK and ERK antibodies. β-actin was used as a loading control.

In a clinical perspective, it is widely acknowledged that paradoxical ERK activation by RAF inhibitors in non-BRAF-mutated cancer cells mostly relies on BRAF and CRAF dimerization. Our model provided the unique opportunity to test the effects of Vemurafenib on NRAS-mutated cells expressing only the ARAF protein, in the complete absence of BRAF and CRAF (Fig. 7). Interestingly, Vemurafenib treatment increased both the intrinsic kinase activity of endogenous ARAF (Fig. 7b) and ERK activation (Fig. 7a,b) in double BRAF- and CRAF-deficient resistant cultures, demonstrating that ARAF homodimers are sufficient to sustain ERK paradoxical activation by BRAF inhibitor in the absence of other RAF proteins.

## Discussion

Aberrant RAS activation plays causal role in human cancer with an estimated 30% of human tumours harbouring somatic oncogenic mutations mainly in KRAS, but also in NRAS and HRAS. NRAS mutations occur in ∼20% of human cutaneous melanomas, while HRAS and KRAS mutations are rare in this disease^2^. NRAS mutations represent an early event in the oncogenic process since 15–20% of benign common nevi and more than 80% of congenital nevi are found mutated^35^. In *Tyr::NRAS*^*Q61K*^*/*^*o*^ transgenic mice, constitutive expression of an activated human *NRAS* allele in melanocytes results in early benign lesions characterized by a hyperpigmented skin and appearance of nevi that can eventually progress to cutaneous melanoma on an *Ink4a*-deficient background^28^,^32^. This model enabled us to evaluate the respective contribution of BRAF and/or CRAF from initiation of melanoma to tumour maintenance by using a conditional approach.

Concomitant loss of BRAF and CRAF in melanocytic lineage completely reversed the hyperpigmented phenotype without affecting the normal development of melanocytes and prevented tumour onset. Likewise, once melanoma have developed, tamoxifen-induced deletion of both kinases completely blocked tumour growth *in vivo* and cell proliferation *in* vitro, the latter being associated with a strong impairment in ERK activation and cell cycle arrest. Other RAS downstream signalling pathways such as RAL and RAC1 have been also implicated in NRAS-driven melanoma^17^,^33^. The role of the PI3K/AKT pathway is less well characterized. NRAS mutant melanomas rarely harbour either mutations in, or silencing of the negative regulator of the PI3K pathway, phosphatase and tensin homologue and exhibit lower levels of constitutive AKT signalling than those with BRAF mutations^36^. Whatever the level of contribution of these signalling pathways in RAS-induced melanoma, our results demonstrate that the RAF/MEK/ERK pathway is absolutely required downstream of NRAS^Q61K^ from initiation to tumour maintenance. Interestingly, resistant colonies emerged in the absence of BRAF and CRAF expression following tamoxifen treatment of melanoma cell cultures. These cells displayed normal levels of ERK activation compared to parental culture and their proliferation was dependent on the MAPK–ERK pathway. We demonstrated that ARAF, which had never been involved in melanoma so far, was responsible for MAPK activation and cell proliferation. This mechanism of resistance not only further supports the critical role of RAF signalling pathway in melanoma, but also highlights its high plasticity in these tumours, which results from the compensatory functions of RAF kinases. Similar compensatory effects were also observed during the establishment of resistance to RAF inhibitors in BRAF-mutated melanoma, with a switch from BRAF to CRAF dependency^37^.

The role of ARAF in NRAS-induced melanoma has not been a matter of intense investigation mainly because of the well-described paradoxical effects of RAF inhibitors in RAS-mutated tumours, which is thought to rely mostly on BRAF and CRAF dimerization. We show here that Vemurafenib was capable of activating both ARAF and ERK in double BRAF- and CRAF-deficient cultures, demonstrating that ARAF homodimers are sufficient to sustain ERK paradoxical activation in the absence of other RAF proteins. Similar observations were recently made in human cell lines from various cancers, using RNA interference^7^,^38^,^39^,^40^. The potential role of ARAF in NRAS-induced melanoma is further reinforced by an *in silico* search in public databases that allowed us to identify patients with metastatic melanomas harbouring an ARAF mutation associated with activating NRAS mutations (Supplementary Fig. 8)^41^,^42^. This activating ARAF^S214F^ mutation has been very recently identified in lung adenocarcinomas^43^,^44^, but to our knowledge, this is the first time that it is detected in melanoma and found specifically associated with an NRAS mutation. Taken together, these findings suggest that ARAF might be also an important player in NRAS-mutated melanoma under specific conditions.

We recently reported similar compensatory effects of RAF kinases in the normal melanocytic lineage homeostasis. Double BRAF and CRAF KO but not single KO mice develop a progressive hair-greying phenotype because of a defect in melanocyte stem cell self-renewal in the hair follicle^21^. In contrast, BRAF and CRAF were not required for melanocyte lineage development. Interestingly, although concomitant ablation of BRAF and CRAF in NRAS^Q61K^-expressing mice reverted the hyperpigmented phenotype, these animals developed a normal coat colour phenotype during the first 3 weeks of life and then progressive hair-greying. Thus, the complete reversion of the NRAS^Q61K^ phenotype was not due to a melanocyte stem cell maintenance defect, since hyperpigmentation phenotype preceded the first melanocyte renewal from stem cell pool of the hair follicle. Taken together, these observations confirm that RAF signalling is dispensable for normal early melanocyte development but absolutely required under pathological conditions triggered by oncogenic RAS.

While recent mouse studies reported a critical role for CRAF in the development of RAS-induced tumours^2^,^9^,^24^,^25^,^26^,^45^,^46^, our models allowing selective and temporal-dependent inactivation of *Raf* genes demonstrated that RAF proteins play both compensatory and specific functions during cutaneous melanoma progression. Indeed, we showed that BRAF but not CRAF plays a critical role during the initiating stages of melanoma formation. Early BRAF depletion resulted in a complete reversion of the hyperpigmented phenotype induced by NRAS. Melanocytes displayed normal skin localization and neither nevi, nor tumours developed from BRAF-deficient animals. In contrast, CRAF appeared dispensable since the phenotype of single BRAF KOs was comparable to that of double BRAF and CRAF KOs. That CRAF could not compensate for BRAF absence is explained by its lower ability to bind NRAS and to activate ERK during early melanomagenesis. Single CRAF KO mice developed, however, a weakly altered hyperpigmented phenotype that could be explained by the fact that BRAF/CRAF heterodimers are more active than BRAF homodimers^47^. Taken together, our results demonstrate that only BRAF is necessary during the early stages of NRAS-induced melanomagenesis, thereby revealing a specific function for BRAF in tumour initiation that cannot be compensated by ARAF and CRAF. This requirement for BRAF activity is strictly restricted to pathological conditions, since the single BRAF KO has otherwise no effect on either development of the melanocyte lineage or melanocyte stem cell self-maintenance^21^.

Using tamoxifen-controlled *Raf* gene recombination, we were also able to analyse the respective contribution of BRAF and CRAF in melanoma growth and maintenance. CreERT2-induced floxed *Braf* allele recombination was efficiently achieved in primary tumours grafted in nude mice or cultured *in vitro*. However, neither slowing down of tumour growth and cell proliferation was evidenced in these experiments. This indicated that BRAF was dispensable after melanoma initiation for tumour growth and maintenance. In grafted tumours and in cell cultures, complete loss of CRAF expression could be achieved and induced a weak but reproducible slowing down of tumour growth and cell proliferation. Altogether, these results suggested that CRAF could take over BRAF to maintain MAPK activity when melanocytic tumours progressed towards malignancy. These observations were in agreement with the existence of a signalling switch from BRAF to CRAF in NRAS-transformed melanocytes^9^. However, the effects of single CRAF deletion were very weak compared to that of the double BRAF/CRAF KO, and far from sufficient to block tumour growth, clearly indicating a compensatory effect implicating BRAF. At the mechanistic level, BRAF was capable of binding to NRAS in both parental and CRAF KO cells, but its kinase activity was notably increased in absence of CRAF. Importantly, these observations were not restricted to mouse melanoma, since we also found that BRAF and CRAF cooperated to activate ERK and to sustain proliferation in three different NRAS-mutated human melanoma cell lines, as also observed by Jaiswal *et al*.^15^.

This work enabled us to propose a model highlighting the respective roles of RAF kinases during NRAS-induced melanoma progression. During the early initiation steps, only BRAF is necessary and neither CRAF, nor ARAF can compensate for BRAF deficiency. After progression to malignancy, both BRAF and CRAF can contribute to tumour maintenance, although BRAF becomes dispensable when CRAF is expressed. Finally, in absence of both BRAF and CRAF, ARAF can eventually be recruited to sustain cell proliferation.

A differential contribution of RAF kinases was also reported in KRAS-driven non-small cell lung carcinoma. BRAF depletion did not affect tumour burden, whereas CRAF was required for tumour initiation^24^,^26^. Another mouse model of RAS-driven skin carcinogenesis revealed that BRAF or CRAF ablation in keratinocytes prevented tumoral initiation and maintenance but through different mechanisms, BRAF being necessary for ERK activation and proliferation and CRAF for inhibition of keratinocyte differentiation^25^,^46^. It would be therefore interesting to understand why RAF kinases differentially contribute to tumorigenesis in these models. Regarding melanoma, our findings establish that BRAF not only plays an obvious key role in BRAF-mutated tumours, but is also essential for NRAS-induced tumours. Altogether, these studies highlight the addiction to the RAF/MAPK pathway in RAS-induced tumorigenesis and reinforce the notion that future improvement of patient treatments will require the development of potent therapeutic agents targeting the overall pathway.

## Methods

### Mice

*Braf*^f/f^ (ref. 48), *Craf*^f/f^ (ref. 49) and *Ink4a*^+/−^ (ref. 50) mice in pure SV129 genetic background and *Tyr::NRAS*^Q61K^/^o^ (ref. 28), *Tyr::Cre*/^o^ (ref. 30), *Tyr::CreERT2*/^o^ (ref. 29) and *Dct::LacZ*/^o^ (ref. 31) mice in pure C57BL6 genetic background were previously described. The strains were interbred to obtain a non-inducible or inducible deletion of BRAF and/or CRAF specifically in the melanocytic lineage. Genotyping was performed as reported^21^,^28^,^29^. As the *Tyr::Cre* transgene is located on the X chromosome, only *Cre*-positive males were used for experimental analyses. Control and wild-type animals refer to *NRAS*-positive and *Cre*-negative or *NRAS*-negative and *Cre*-negative mice, respectively. Every 2 weeks, mice were analysed macroscopically after shaving for the occurrence of tumours and morbidity signs. Depilation experiments were described previously^21^. For FACS-sorting experiments of GFP-positive melanocytes, *Tyr::NRAS*^Q61K^/^o^*;Ink4a*^+/−^;*Tyr::Cre/*^o^ mice were crossed with the *mT/mG* mice^34^ that express membrane-targeted GFP after Cre-mediated excision. Experimental procedures were conducted in accordance with recommendations of the European Union (86/609/EEC) and the French National Committee (87/848). Animal care and use were approved by the ethics committee of the Curie Institute in compliance with the institutional guidelines.

### Histological analyses and immunostaining

Haematoxylin and eosin staining on paraffin-embedded skin sections or X-Gal staining on frozen skin sections were described previously^21^. P-ERK immunostaining was performed on 6 μm frozen sections from P10 skins fixed in 4% paraformaldehyde. Permeabilized sections were boiled in10 mM sodium citrate, treated with blocking solution (10% goat serum in PBS/0.1% Tween20) and incubated with primary antibodies: chicken anti-βGal (1:300; #9361; Abcam) and rabbit anti-phospho-ERK (1:100; #4376; Cell Signaling). Alexa Fluor 488 or Alexa Fluor 594 (Invitrogen) was used as secondary antibodies.

### Tumour transplantation and *in vivo* tamoxifen treatment

The tumour was collected from the donor animal, cut into small pieces (5 × 5 mm) and transplanted subcutaneously in the right flank of two cohorts of seven or eight nude mice (6-week-old Swiss Nu/Nu females, Charles River Laboratories). One week later, mice received daily an intraperitoneal injection of tamoxifen (Sigma T5648, 1 mg per mouse, 10 mg ml^−1^ in EtOH 95%/corn oil 5%) or vehicle for 10 days. Tumour growth was measured every week for up to 6 weeks. At the end of the experiment, mice were killed and tumours were collected. Total lysates were prepared in lysis buffer (Tris 20 mM pH 7.4, NaCl 100 mM, Triton X-100 0.5%, protease and phosphatase inhibitors) using Precellys tissue homogenizer.

### Cell culture experiments

NRAS-mutated human melanoma cell lines (WM1361, WM852 and Sbcl2 from M. Herlyn'lab) were cultured in RPMI 1640 medium (GIBCO) supplemented with 10% fetal bovine serum (FBS), penicillin and streptomycin. Cells were tested for mycoplasma contamination and were cultured at 37 °C in a humidified atmosphere containing 5% CO_2_. For growth curves, 5 × 10^4^ murine or human melanoma cell lines were seeded into six-well plates and counted after staining with Trypan blue by using a LUNA cell counter (LogoBiosystems). When indicated, cells were treated with 4OHT (500 nM, Sigma), U0126 MEK inhibitor (10 μM, Sigma) or Vemurafenib (PLX4032; 1 μM, Selleckchem). Cell extracts were prepared in lysis buffer (Tris 20 mM pH 7.4, NaCl 100 mM, Triton X-100 0.5%, protease and phosphatase inhibitors). Immunoprecipitation experiments were performed on 5 × 10^6^ cells with 25 μl of anti-NRAS antibody and 20 μl of a 50% slurry of protein A-Sepharose (GE Healthcare). For *in vitro* kinase assays, ARAF, BRAF or CRAF were immunoprecipitated (#4432, Cell Signaling; sc5284, Santa Cruz; #610151, BD Biosciences, , San Jose, CA, respectively) and the immune complexes were incubated with 1 μg of purified kinase inactive GST-MEK (Millipore) in 30 mM Tris (pH 7.5), 0.1 mM EDTA, 10 mM MgCl_2_, 0.1% Triton X-100, 5 mM NaF, 1 mM ATP, 0.3% β-mercaptoethanol for 30 min at 30 °C. The reaction was analysed by SDS–PAGE with phospho-MEK (#2336, Cell Signaling Technology), ARAF, BRAF or CRAF antibodies.

### Establishment of melanoma cell lines from primary tumours

Primary tumour tissue was dissected from melanoma-bearing *Braf*^+/+^*;Craf*^f/f^*;Tyr::NRAS*^Q61K^/^o^*;Ink4a*^+/−^*;Tyr::CreERT2*/^o^, *Braf*^f/f^*;Craf*^+/+^*;Tyr::NRAS*^Q61K^/^o^*;Ink4a*^+/−^*;Tyr::CreERT2*/^o^ or *Braf*^f/f^*;Craf*^f/f^*; Tyr::NRAS*^Q61K^/^o^*;Ink4a*^+/−^*;Tyr::CreERT2*/^o^ mice and cut into small pieces. Samples were treated with 0.25% (w/v) of type I and IV collagenases (Sigma) in PBS at 37 °C for 45 min, washed with Wash Buffer (Hank's balanced salt solution with 1 mM CaCl_2_, 0.005% DNase I (Roche), 20% FBS (Sigma)) and incubated in Cell Dissociation Buffer (GIBCO/Invitrogen) at 37 °C for 10 min. The skin was dissociated by passing through 100 μm cell strainer and 18–20 gauge needles. The cells were plated onto 6 wells-plates (1 × 10^6^ cells per well) and cultured in HAM F-12 Medium (GIBCO/Invitrogen) containing 10% FBS, 100 units ml^−1^ penicillin, 100 μg ml^−1^ streptomycin and 2 mM l-glutamine (Invitrogen).

### Western blotting and antibodies

For SDS–PAGE analysis, the membranes were blocked with 5% low-fat milk in PBS Tween 20 (10%) for 30 min at room temperature. Membranes were then probed overnight at 4 °C with the appropriated primary antibodies: anti-ARAF (sc408, Santa Cruz, 1:500), anti-BRAF (sc5284, Santa Cruz, 1:2,000), anti-CRAF (#610151, BD Biosciences, 1:2,000), anti-NRAS (sc519, Santa Cruz, 1:2,000), anti-pMEK (#9121, Cell Signaling, 1:1,000), anti-MEK (sc219, Santa Cruz, 1:2,000), anti-pERK (M8159, Sigma, 1:2,000), anti-ERK (sc93, Santa Cruz, 1:2,000) and anti-βactin (A1978, Sigma, 1:5,000) antibodies. Signals were acquired using a CDD camera (G:BOX, Syngene). Uncropped western blotting pictures are shown in Supplementary Fig. 9.

### Cell-sorting experiments by FACS

Whole skin was harvested from *Tyr::NRAS*^Q61K^/^o^*;Ink4a*^+/−^;*Tyr::Cre/*^o^;*mT/mG* mice at P10 and digested in a mixture of dispase (Invitrogen) and collagenase IV (Sigma) for 2 h 30 min at 37 °C and then mechanically dissociated with forceps followed by multiple passages trough an 18-G needle. Samples were strained in FACS buffer (RPMI, 5% fetal calf serum (FCS), 2 mM EDTA) in order to obtain single-cell suspensions and counterstained with anti-p75 NGF Receptor antibody (ab8874, Abcam, 1:500). Cell sorting was performed on a FACS-AriaIII (BD Biosciences). GFP+, p75− cells were plated on vitronectin-coated glass coverslips in HAM F12 medium conditioned on a feeder of human keratinocytes. Twenty-four hours later, cells were fixed in paraformaldehyde (PFA) 4% and permeabilized in Tween 20 0.2%.

### Proximity ligation assay

Cells were grown on glass coverslips, fixed and permeabilized. PLA (Duolink) was performed according to the manufacturer's (Olink) instructions using antibodies against BRAF (sc5284, Santa Cruz, 1:500) and NRAS (sc519, Santa Cruz, 1:50), or CRAF (#610151, BD Biosciences, 1:50) and NRAS. Knockout cells for BRAF or CRAF were used as control. Images were captured using a 3D/optigrid Leica fluorescent microscope. The average number of dots per cell (identified by its nucleus) was determined by analysing at least 70 different cells with the ImageJ software.

### Lentiviral production and infection

Lentiviral pLKO vectors encoding shRNA control, targeting murine ARAF (NM_9703, clones TRCN0000287252 for shARAF.1, TRCN0000307404 for shARAF.2, TRCN0000294819 for shARAF.3) were obtained from Sigma (Mission shRNA library). Lentiviruses were produced in 293T cells, by co-transfecting pLKO-derived vectors and the packaging plasmids pS-PAX2 and pMD2-VSVG, using lipofectamine 2000 (Invitrogen). Lentiviral particles were harvested 48 h post transfection. Murine melanoma cell lines were seeded into six-well plates (10^5^ cells per well) and infected with 100 μl of viral supernatant in 700 μl of medium. The virus was replaced with fresh medium after 48 h and selection (puromycine 1 μg ml^−1^ or neomycin 2 mg ml^−1^, respectively) was applied. Stable established cell lines were either passed or stained with crystal violet.

### *In vitro* siRNA experiments and BrdU labelling

Cells were seeded at 8 × 10^4^ cells per well in 12-well plates and transfected with 80 pmol of BRAF-specific, CRAF-specific or control scrambled siRNA using LipofectAMINE (Gibco). Twenty-four hours later, cells were either harvested or BrdU-labelled. The siRNA sequences used were as follow: siBRAF1 5′-AAGUGGCAUGGUGAUGUGGCA-3′; siBRAF2 5′-AGAAUUGGAUCUGGAUCAU-3′; siCRAF1 5′-GCACGCUUAGAUUGGAAUA-3′; siCRAF2 5′-AAUAGUUCAGCAGUUUGGCUA-3′; scrambled ScrSi 5′-AAGUCCAUGGUGACAGGAGAC-3′ (refs 39, 51). For BrdU labelling, after BrdU incorporation (20 μM), cells were fixed with 4% formaldehyde, permeabilized and blocked in 0.2% Triton X-100, 10% FCS in PBS and stained (1:500 anti-BrdU antibody (Sigma), 0.5 mg ml^−1^ DNase I). Donkey anti-mouse Alexa Fluor 594 (Invitrogen) was used for detection. Counterstaining with 4,6-diamidino-2-phenylindole (DAPI) was performed to quantify BrdU-positive cells.

### Cell cycle analyses by flow cytometry

On day 6 of 4OHT treatment, cells were incubated for 30 min with BrdU (final concentration 10 μM). Cells were labelled for BrdU incorporation with an FITC BrdU flow kit (BD Pharmingen), according to the manufacturer's protocol and resuspended in 300 ml of PBS containing 20 ml of 7-AAD. The signals were detected by FACScalibur cytometer (BD Biosciences) and analysed using the FlowJo software.

### Real-time RT–PCR

Total RNAs were extracted using the RNeasy Plus mini Kit (Qiagen) and reversely transcribed with the Cloned AMV First-Strand cDNA Synthesis Kit (Invitrogen). Quantitative real-time PCR assays were conducted using SYBR Green real-time PCR Master Mix and real-time PCR amplification equipment (Applied Biosystem; forward primer, 5′-GAAGACAAGCCCAAGATGGA-3′; reverse prime, 5′-CTCAGCCCCACTAACAGGAG-3′).

### Data availability

The data that support the findings of this study are available from the corresponding authors on request.

## Additional information

**How to cite this article:** Dorard, C. *et al*. RAF proteins exert both specific and compensatory functions during tumor progression of NRAS-driven melanoma. *Nat. Commun.* **8,** 15262 doi: 10.1038/ncomms15262 (2017).

**Publisher's note:** Springer Nature remains neutral with regard to jurisdictional claims in published maps and institutional affiliations.

## Supplementary Material

Supplementary Information Supplementary Figures

## References

[b1] Miller A. J. & Mihm M. C. Melanoma. N. Engl. J. Med. 355, 51–65 (2006). 16822996 10.1056/NEJMra052166

[b2] Fedorenko I. V., Gibney G. T. & Smalley K. S. M. NRAS mutant melanoma: biological behavior and future strategies for therapeutic management. Oncogene 32, 3009–3018 (2013). 23069660 10.1038/onc.2012.453 PMC3978385

[b3] Garnett M. J. & Marais R. Guilty as charged: B-RAF is a human oncogene. Cancer Cell 6, 313–319 (2004). 15488754 10.1016/j.ccr.2004.09.022

[b4] Chapman P. B. . Improved survival with vemurafenib in melanoma with BRAF V600E mutation. N. Engl. J. Med. 364, 2507–2516 (2011). 21639808 10.1056/NEJMoa1103782 PMC3549296

[b5] Hauschild A. . Dabrafenib in BRAF-mutated metastatic melanoma: a multicentre, open-label, phase 3 randomised controlled trial. Lancet 380, 358–365 (2012). 22735384 10.1016/S0140-6736(12)60868-X

[b6] Lito P., Rosen N. & Solit D. B. Tumor adaptation and resistance to RAF inhibitors. Nat. Med. 19, 1401–1409 (2013). 24202393 10.1038/nm.3392

[b7] Hatzivassiliou G. . RAF inhibitors prime wild-type RAF to activate the MAPK pathway and enhance growth. Nature 464, 431–435 (2010). 20130576 10.1038/nature08833

[b8] Heidorn S. J. . Kinase-dead BRAF and oncogenic RAS cooperate to drive tumor progression through CRAF. Cell 140, 209–221 (2010). 20141835 10.1016/j.cell.2009.12.040 PMC2872605

[b9] Dumaz N. . In melanoma, RAS mutations are accompanied by switching signaling from BRAF to CRAF and disrupted cyclic AMP signaling. Cancer Res. 66, 9483–9491 (2006). 17018604 10.1158/0008-5472.CAN-05-4227

[b10] Kannengiesser C. . Gene expression signature associated with BRAF mutations in human primary cutaneous melanomas. Mol. Oncol. 1, 425–430 (2008). 19383316 10.1016/j.molonc.2008.01.002 PMC5543835

[b11] Solit D. B. . BRAF mutation predicts sensitivity to MEK inhibition. Nature 439, 358–362 (2006). 16273091 10.1038/nature04304 PMC3306236

[b12] Albino A. P., Le Strange R., Oliff A. I., Furth M. E. & Old L. J. Transforming ras genes from human melanoma: a manifestation of tumour heterogeneity? Nature 308, 69–72 (1984). 6700714 10.1038/308069a0

[b13] Milagre C. . A mouse model of melanoma driven by oncogenic KRAS. Cancer Res. 70, 5549–5557 (2010). 20516123 10.1158/0008-5472.CAN-09-4254 PMC2896549

[b14] Gray-Schopfer V., Wellbrock C. & Marais R. Melanoma biology and new targeted therapy. Nature 445, 851–857 (2007). 17314971 10.1038/nature05661

[b15] Jaiswal B. S. . Combined targeting of BRAF and CRAF or BRAF and PI3K effector pathways is required for efficacy in NRAS mutant tumors. PLoS ONE 4, e5717 (2009). 19492075 10.1371/journal.pone.0005717 PMC2683562

[b16] Krauthammer M. . Exome sequencing identifies recurrent somatic RAC1 mutations in melanoma. Nat. Genet. 44, 1006–1014 (2012). 22842228 10.1038/ng.2359 PMC3432702

[b17] Mishra P. J. . Dissection of RAS downstream pathways in melanomagenesis: a role for Ral in transformation. Oncogene 29, 2449–2456 (2010). 20118982 10.1038/onc.2009.521 PMC3287039

[b18] Kos L. . Hepatocyte growth factor/scatter factor-MET signaling in neural crest-derived melanocyte development. Pigment Cell Res. 12, 13–21 (1999). 10193678 10.1111/j.1600-0749.1999.tb00503.x

[b19] Wu M. . c-Kit triggers dual phosphorylations, which couple activation and degradation of the essential melanocyte factor Mi. Genes Dev. 14, 301–312 (2000). 10673502 PMC316361

[b20] Peyssonnaux C. & Eychène A. The Raf/MEK/ERK pathway: new concepts of activation. Biol. Cell 93, 53–62 (2001). 11730323 10.1016/s0248-4900(01)01125-x

[b21] Valluet A. . B-Raf and C-Raf are required for melanocyte stem cell self-maintenance. Cell Rep. 2, 774–780 (2012). 23022482 10.1016/j.celrep.2012.08.020

[b22] Holderfield M. . RAF inhibitors activate the MAPK pathway by relieving inhibitory autophosphorylation. Cancer Cell 23, 594–602 (2013). 23680146 10.1016/j.ccr.2013.03.033

[b23] Poulikakos P. I., Zhang C., Bollag G., Shokat K. M. & Rosen N. RAF inhibitors transactivate RAF dimers and ERK signalling in cells with wild-type BRAF. Nature 464, 427–430 (2010). 20179705 10.1038/nature08902 PMC3178447

[b24] Blasco R. B. . c-Raf, but not B-Raf, is essential for development of K-Ras oncogene-driven non-small cell lung carcinoma. Cancer Cell 19, 652–663 (2011). 21514245 10.1016/j.ccr.2011.04.002 PMC4854330

[b25] Ehrenreiter K. . Raf-1 addiction in Ras-induced skin carcinogenesis. Cancer Cell 16, 149–160 (2009). 19647225 10.1016/j.ccr.2009.06.008

[b26] Karreth F. A., Frese K. K., DeNicola G. M., Baccarini M. & Tuveson D. A. C-Raf is required for the initiation of lung cancer by K-Ras(G12D). Cancer Discov. 1, 128–136 (2011). 22043453 10.1158/2159-8290.CD-10-0044 PMC3203527

[b27] Davies H. . Mutations of the BRAF gene in human cancer. Nature 417, 949–954 (2002). 12068308 10.1038/nature00766

[b28] Ackermann J. . Metastasizing melanoma formation caused by expression of activated N-RasQ61K on an INK4a-deficient background. Cancer Res. 65, 4005–4011 (2005). 15899789 10.1158/0008-5472.CAN-04-2970

[b29] Yajima I. . Spatiotemporal gene control by the Cre-ERT2 system in melanocytes. Genesis 44, 34–43 (2006). 16419042 10.1002/gene.20182

[b30] Delmas V., Martinozzi S., Bourgeois Y., Holzenberger M. & Larue L. Cre-mediated recombination in the skin melanocyte lineage. Genesis 36, 73–80 (2003). 12820167 10.1002/gene.10197

[b31] Mackenzie M. A., Jordan S. A., Budd P. S. & Jackson I. J. Activation of the receptor tyrosine kinase Kit is required for the proliferation of melanoblasts in the mouse embryo. Dev. Biol. 192, 99–107 (1997). 9405100 10.1006/dbio.1997.8738

[b32] Campagne C. . Histopathological atlas and proposed classification for melanocytic lesions in Tyr::NRas(Q61K); Cdkn2a(−/−) transgenic mice. Pigment Cell Melanoma Res. 26, 735–742 (2013). 23647911 10.1111/pcmr.12115

[b33] Li A. . Activated mutant NRas(Q61K) drives aberrant melanocyte signaling, survival, and invasiveness via a Rac1-dependent mechanism. J. Invest. Dermatol. 132, 2610–2621 (2012). 22718121 10.1038/jid.2012.186 PMC3472562

[b34] Muzumdar M. D., Tasic B., Miyamichi K., Li L. & Luo L. A global double-fluorescent Cre reporter mouse. Genesis 45, 593–605 (2007). 17868096 10.1002/dvg.20335

[b35] Bauer J., Curtin J. A., Pinkel D. & Bastian B. C. Congenital melanocytic nevi frequently harbor NRAS mutations but no BRAF mutations. J. Invest. Dermatol. 127, 179–182 (2007). 16888631 10.1038/sj.jid.5700490

[b36] Davies M. A. . Integrated molecular and clinical analysis of AKT activation in metastatic melanoma. Clin. Cancer Res. 15, 7538–7546 (2009). 19996208 10.1158/1078-0432.CCR-09-1985 PMC2805170

[b37] Montagut C. . Elevated CRAF as a potential mechanism of acquired resistance to BRAF inhibition in melanoma. Cancer Res. 68, 4853–4861 (2008). 18559533 10.1158/0008-5472.CAN-07-6787 PMC2692356

[b38] Mooz J. . Dimerization of the kinase ARAF promotes MAPK pathway activation and cell migration. Sci. Signal. 7, ra73 (2014). 25097033 10.1126/scisignal.2005484

[b39] Rebocho A. P. & Marais R. ARAF acts as a scaffold to stabilize BRAF:CRAF heterodimers. Oncogene 32, 3207–3212 (2013). 22926515 10.1038/onc.2012.330

[b40] Kaplan F. M., Shao Y., Mayberry M. M. & Aplin A. E. Hyperactivation of MEK-ERK1/2 signaling and resistance to apoptosis induced by the oncogenic B-RAF inhibitor, PLX4720, in mutant N-RAS melanoma cells. Oncogene 30, 366–371 (2011). 20818433 10.1038/onc.2010.408 PMC6591715

[b41] Gao J. . Integrative analysis of complex cancer genomics and clinical profiles using the cBioPortal. Sci. Signal. 6, pl1 (2013). 23550210 10.1126/scisignal.2004088 PMC4160307

[b42] Cerami E. . The cBio cancer genomics portal: an open platform for exploring multidimensional cancer genomics data. Cancer Discov. 2, 401–404 (2012). 22588877 10.1158/2159-8290.CD-12-0095 PMC3956037

[b43] Imielinski M. . Oncogenic and sorafenib-sensitive ARAF mutations in lung adenocarcinoma. J. Clin. Invest. 124, 1582–1586 (2014). 24569458 10.1172/JCI72763 PMC3973082

[b44] Berger A. H. . High-throughput phenotyping of lung cancer somatic mutations. Cancer Cell 30, 214–228 (2016). 27478040 10.1016/j.ccell.2016.06.022 PMC5003022

[b45] Dumaz N. Mechanism of RAF isoform switching induced by oncogenic RAS in melanoma. Small GTPases 2, 289–292 (2011). 22292133 10.4161/sgtp.2.5.17814 PMC3265821

[b46] Kern F., Doma E., Rupp C., Niault T. & Baccarini M. Essential, non-redundant roles of B-Raf and Raf-1 in Ras-driven skin tumorigenesis. Oncogene 32, 2483–2492 (2013). 22733131 10.1038/onc.2012.254

[b47] Rushworth L. K., Hindley A. D., O'Neill E. & Kolch W. Regulation and role of Raf-1/B-Raf heterodimerization. Mol. Cell Biol. 26, 2262–2272 (2006). 16508002 10.1128/MCB.26.6.2262-2272.2006 PMC1430271

[b48] Chen A. P. . Forebrain-specific knockout of B-raf kinase leads to deficits in hippocampal long-term potentiation, learning, and memory. J. Neurosci. Res. 83, 28–38 (2006). 16342120 10.1002/jnr.20703

[b49] Jesenberger V. . Protective role of Raf-1 in Salmonella-induced macrophage apoptosis. J. Exp. Med. 193, 353–364 (2001). 11157055 10.1084/jem.193.3.353 PMC2195927

[b50] Serrano M. . Role of the INK4a locus in tumor suppression and cell mortality. Cell 85, 27–37 (1996). 8620534 10.1016/s0092-8674(00)81079-x

[b51] Wellbrock C. . V599EB-RAF is an oncogene in melanocytes. Cancer Res. 64, 2338–2342 (2004). 15059882 10.1158/0008-5472.can-03-3433

