# Supplementary material for: RAF proteins exert both specific and compensatory functions during tumour progression of NRAS-driven melanoma
Source: Nat Commun. 2017 May 12;8:15262. doi: 10.1038/ncomms15262 (PMC5437303; doi:10.1038/ncomms15262)

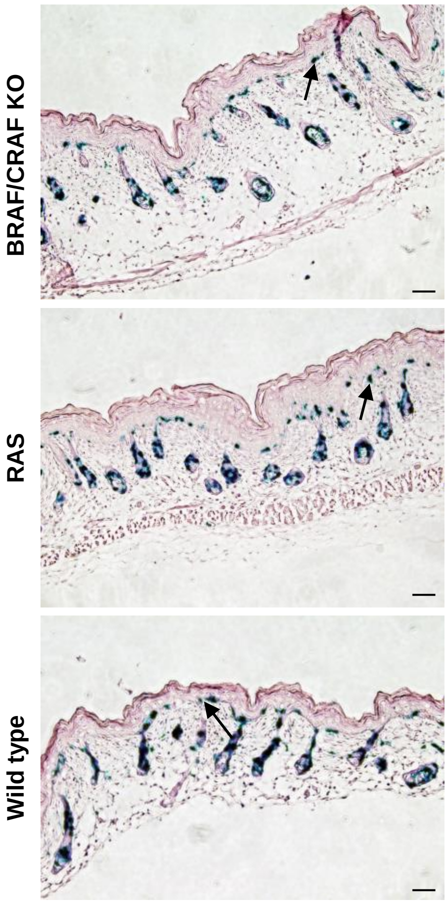

Supplementary Figure 1. X-gal staining of dorsal skin sections from double BRAF/CRAF knockout, RAS control and wild type newborn mice at P0.5. Dermal melanocytes are black arrowed. Scale bar, 500µm.

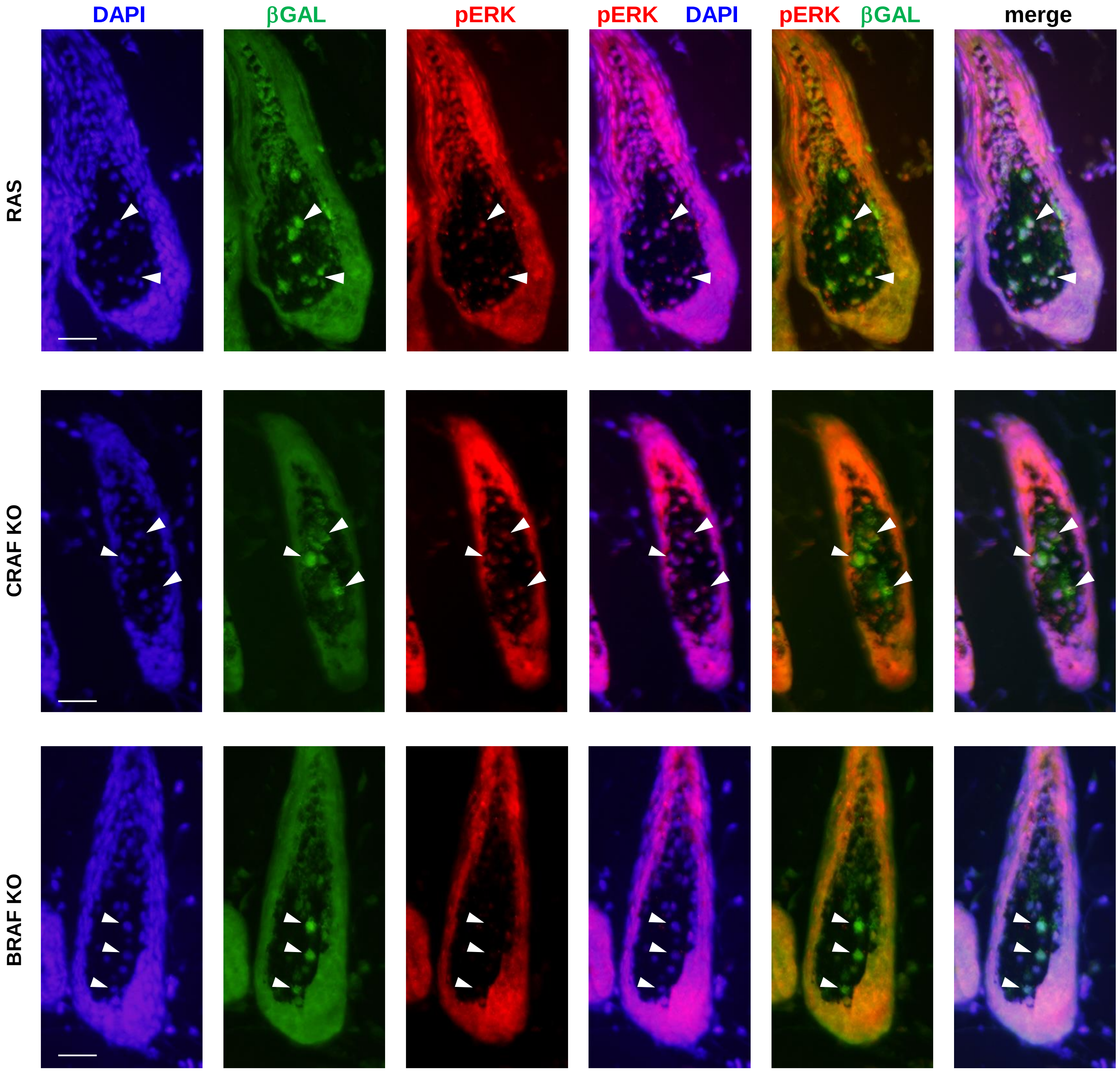

Supplementary Figure 2. Representative examples of immunostaining of hair follicles from RAS control (*Tyr::NRAS*^{Q61K}/^{o}*; Ink4a*$^{+/-}$*; Tyr::Cre/*^{o}*; Dct::LacZ/*^{o}), CRAF KO (*Braf*$^{+/+}$*; Craf*$^{f/f}$*; Tyr::NRAS*^{Q61K}/^{o}*; Ink4a*$^{+/-}$*; Tyr::Cre/*^{o}*; Dct::LacZ/*^{o}) or BRAF KO (*Braf*$^{f/f}$*; Craf*$^{+/+}$*; Tyr::NRAS*^{Q61K}/^{o}*; Ink4a*$^{+/-}$*; Tyr::Cre/*^{o}*; Dct::LacZ/*^{o}) skins at P10. Frozen skin sections were immunolabeled with antiphospho-ERK (pERK) and anti-β-Galactosidase (βGAL) antibodies. βGAL+ melanocytes are indicated with white arrowheads. Scale bar, 25µm. Quantification is shown in Fig. 1g**.**

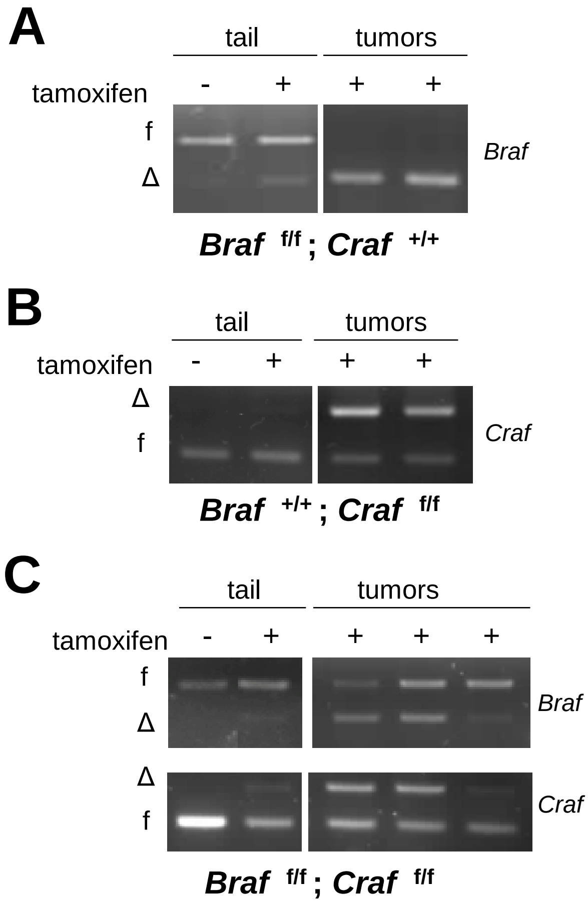

Supplementary Figure 3. PCR analyses of BRAF and/or CRAF recombination in NRAS-induced tumors treated by tamoxifen. Tumors from (A) *Braf*$^{f/f}$*;Craf*$^{+/+}$*;Tyr::NRAS*$^{Q61K/o}$*;Ink4a*$^{+/-}$*;Tyr::CreERT2*$^{/o}$, (B) *Braf*$^{+/+}$*;Craf*$^{f/f}$*;Tyr::NRAS*$^{Q61K/o}$*;Ink4a*$^{+/-}$*;Tyr::CreERT2*$^{/o}$ or (C) *Braf*$^{f/f}$*;Craf*$^{f/f}$*;Tyr::NRAS*$^{Q61K/o}$*;Ink4a*$^{+/-}$*;Tyr::CreERT2*$^{/o}$ mice were either treated daily with intraperitoneal injections (IP) of tamoxifen (1mg per mouse) for 10 days, or received alternately tamoxifen by IP (2.5mg per mouse) and 4-hydroxy-tamoxifen (4OHT) by painting of the tumors (0.1mg per tumor) during 5 days every month. Of note, both protocols gave rise to similar results. When tumor size reached ethical limits, animals were sacrificed and tumoral tissue was collected Recombination efficiency for *Braf* and/or *Craf* was assessed by PCR on DNA either from the tail before and after treatment or from the tumor after treatment. These PCRs are representative of tumors from (A) 4, (B) 7 or (C) 9 different males or females around 8 months-old on a SV129/C57Bl6 mixed genetic background. f indicates PCR products corresponding to *Braf* and *Craf* floxed alleles; Δ indicates PCR products corresponding to *Braf* and *Craf* recombinant alleles.

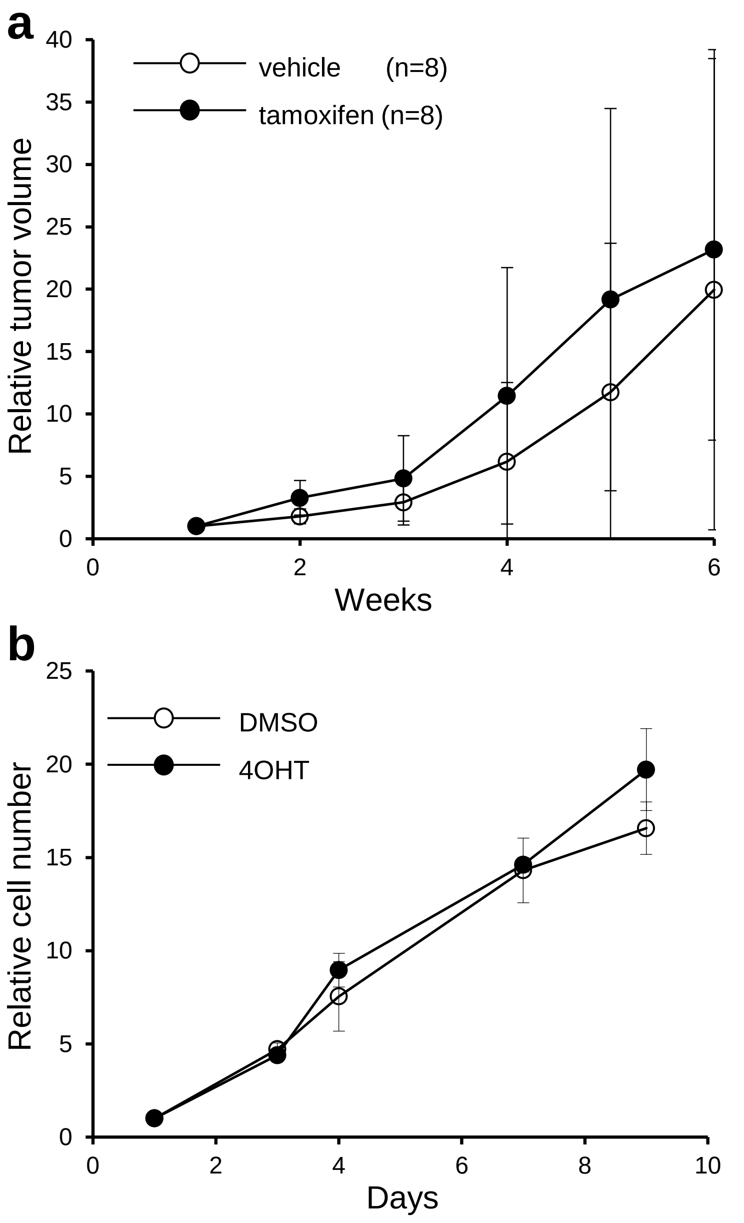

Supplementary Figure 4. Control tumors or melanoma cell lines showing the absence of side effects of treatment on tumor growth or cell proliferation. (a) A melanoma from an untreated *Braf*$^{f/f}$*;Craf*$^{f/f}$*;Tyr::NRAS*$^{Q61K/o}$*;Ink4a*$^{+/-}$ mouse was subcutaneously grafted into two groups of eight nude mice that were treated either with tamoxifen or vehicle for 2 weeks. The effect on tumor growth was assessed by measuring tumor volume over a 6-week period. Data are representative of two independent experiments requiring 32 Swiss Nu/Nu females (6-week-old) for one primary tumor from a 15 months-old female on a SV129/C57Bl6 mixed genetic background. (b) Growth curve analysis of melanoma cell culture established from an untreated *Braf*$^{f/f}$*;Craf*$^{f/f}$*;Tyr::NRAS*$^{Q61K/o}$*;Ink4a*$^{+/-}$ primary mouse tumor in response to 4OHT or DMSO for 9 days. Data are representative of two independent experiments from two cell cultures. All data are represented as mean±s.d.

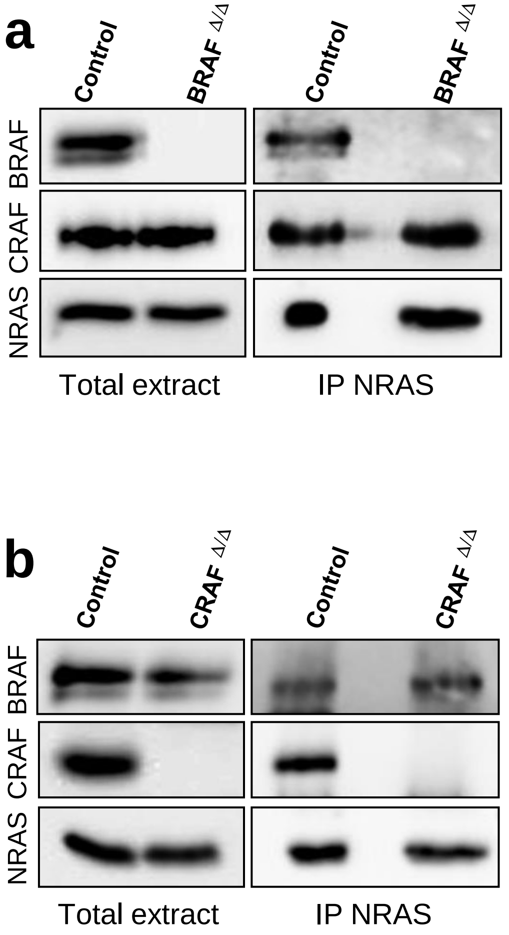

Supplementary Figure 5. Co-immunoprecipitations showing NRAS/BRAF and NRAS/CRAF complexes in BRAF$^{\Delta/\Delta}$ or CRAF$^{\Delta/\Delta}$ compared to control parental cultures (panels a and b, respectively). Cell extracts were immunoprecipitated with anti-NRAS antibody, and immune complexes were immunoblotted with anti-NRAS, anti-BRAF and anti-CRAF antibodies.

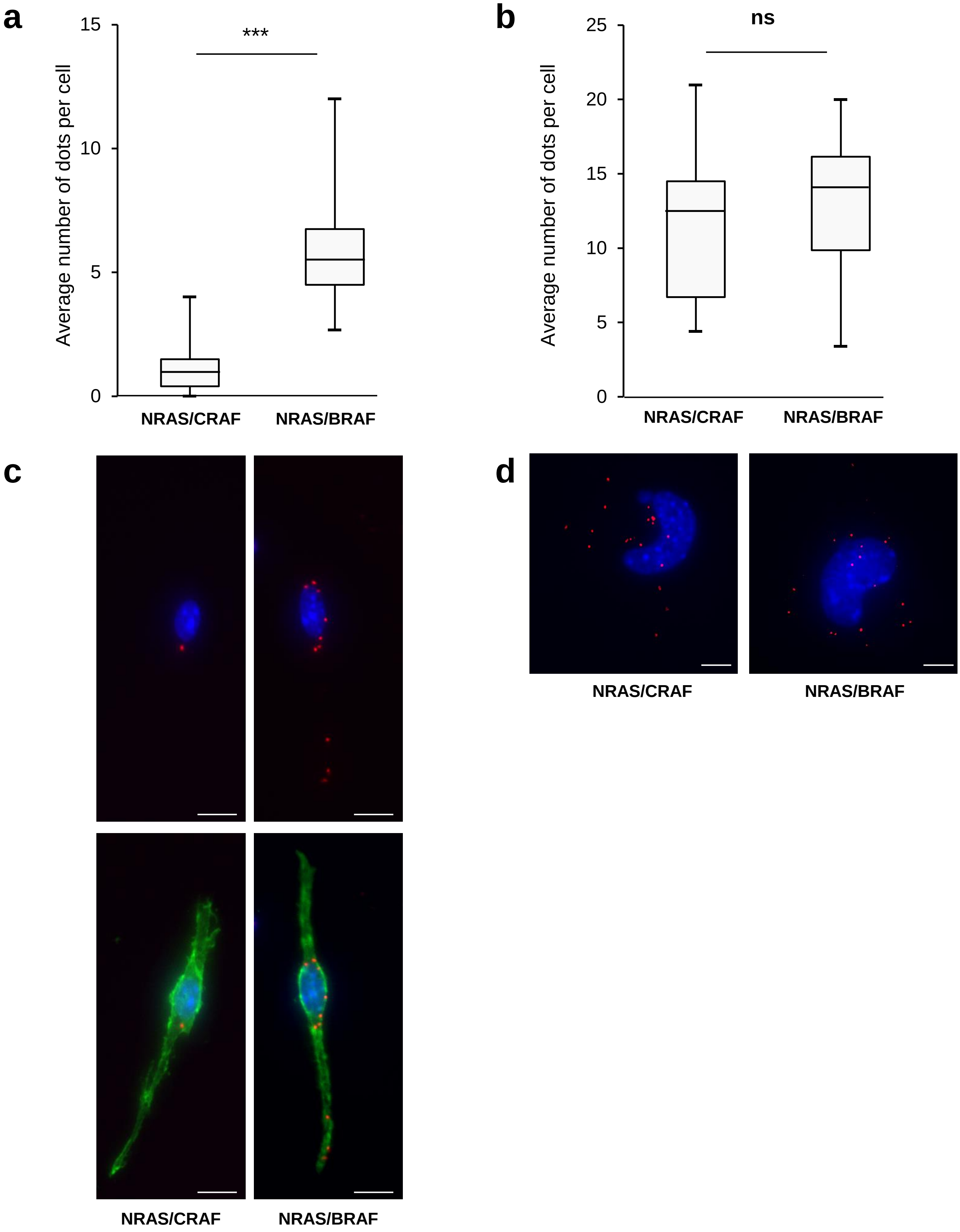

Supplementary Figure 6. NRAS-binding of BRAF and CRAF in NRAS-mutated murine melanocytes compared to NRAS-induced murine melanoma cultures (panels a,c and b,d, respectively)**.** Melanocytes were isolated from the skin of *Tyr::NRAS*^{Q61K}/^{o};*Ink4a*$^{+/-}$;*Tyr::Cre/*^{o};*mT/mG* mice at P10 by cell-sorting FACS experiments. Proximity ligation assays showing NRAS/CRAF and NRAS/BRAF complexes were performed 24h later (c) in comparison to melanoma cultures established from NRAS-mutated control mice (d). Cell nuclei were stained with DAPI. Box plots (a,b respective to c,d) represent the average number of dots per cell. These experiments (a,c) are representative of two P10 males on a SV129/C57Bl6 mixed genetic background. Lower, median, and upper quartiles are shown, with whiskers extending to the lowest and highest values. ***p-value<0.001 compared by t test. Scale bar, 100µm.

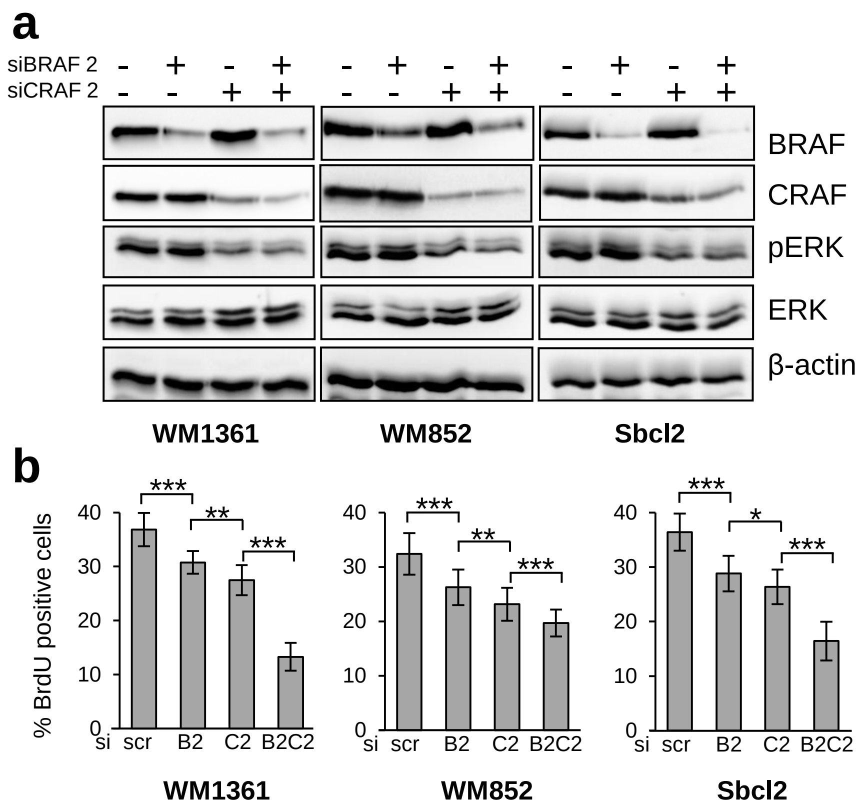

Supplementary Figure 7. Validation of siRNA experiments on human melanoma cell lines by using a second siBRAF2/siCRAF2 couple. (a) Western blot analysis of BRAF and CRAF protein expression levels and pERK activation in three different NRAS mutated human melanoma cell lines (WM1361, WM852 and Sbcl2) transfected with the scrambled control (-) or short interfering RNA to BRAF and/or CRAF (siBRAF2/siCRAF2). (b) BrdU incorporation in WM1361, WM852 and Sbcl2 transfected with control, BRAF, CRAF or BRAF/CRAF siRNA (scr, B2, C2, B2C2 respectively). *p-value<0.05, **p-value<0.01 and ***p-value<0.001 compared by Student's test. All data are represented as mean±s.d.

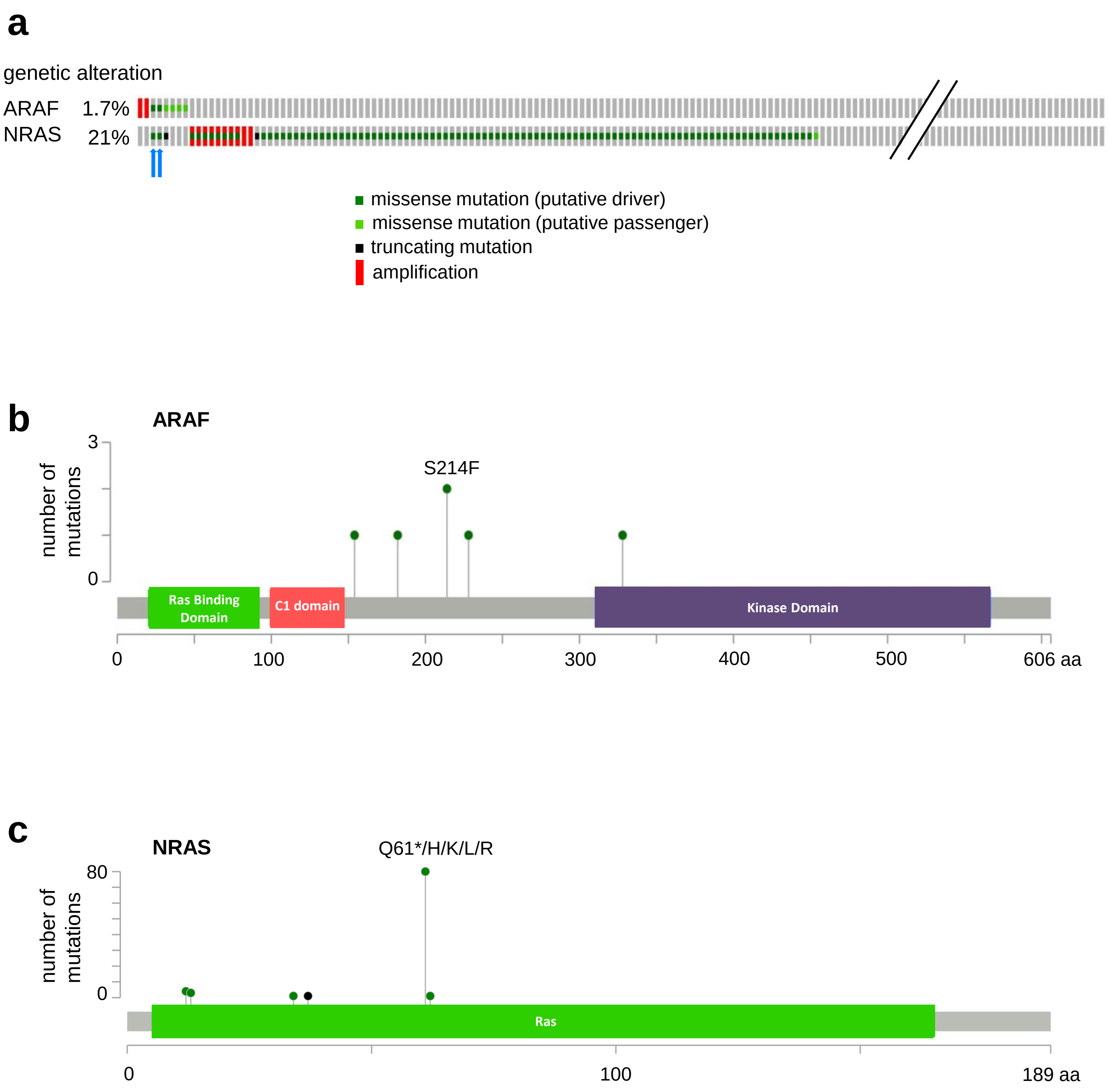

Supplementary Figure 8. Identification of two melanoma patients harboring concomitant ARAF and NRAS activating mutations. (a) Heatmap showing the distribution of mutations in ARAF and NRAS in 471 cutaneous melanoma cases (TCGA Skin Cutaneous Melanoma, cBioPortal) [41, 42]. Each column represents an individual case and each row denotes a specific gene. Dark green indicates the presence of putative driver mutations, light green indicates the presence of putative passenger mutations and light grey refers to the absence of mutation. Blue arrows highlight the two cases where NRAS and ARAF are co-mutated. For reasons of space constraint, the cohort was interrupted by slanting bars but the hidden cases do not show NRAS and ARAF mutations. (b) Location of the identified mutations in the ARAF protein. Green circles represent missense mutations. The most frequent mutation is S214F. (c) Locations of the identified mutations in the NRAS protein. Green circles represent missense mutations and the black circle represents a truncating mutation. The most frequent mutation is located at Q61*.

Supplementary Figure 9. Uncropped western blotting pictures.

Figure 2b

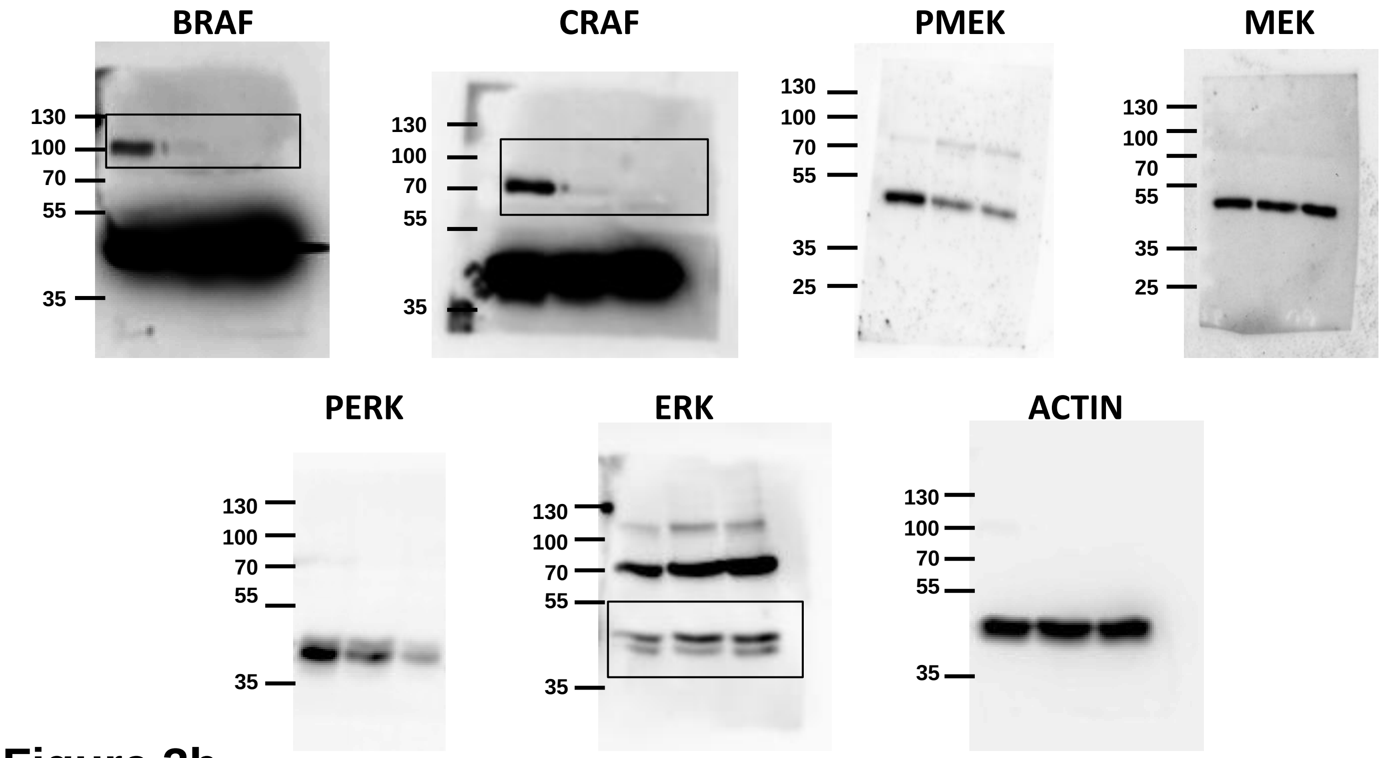

Figure 3b

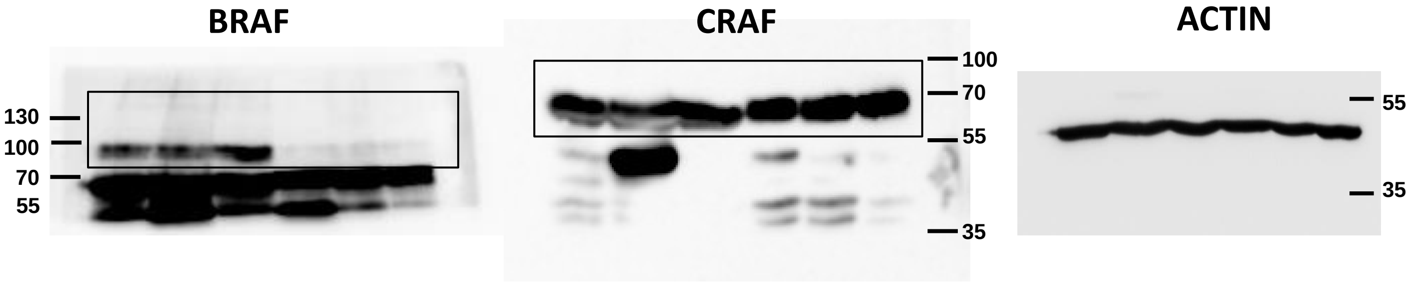

Figure 3d

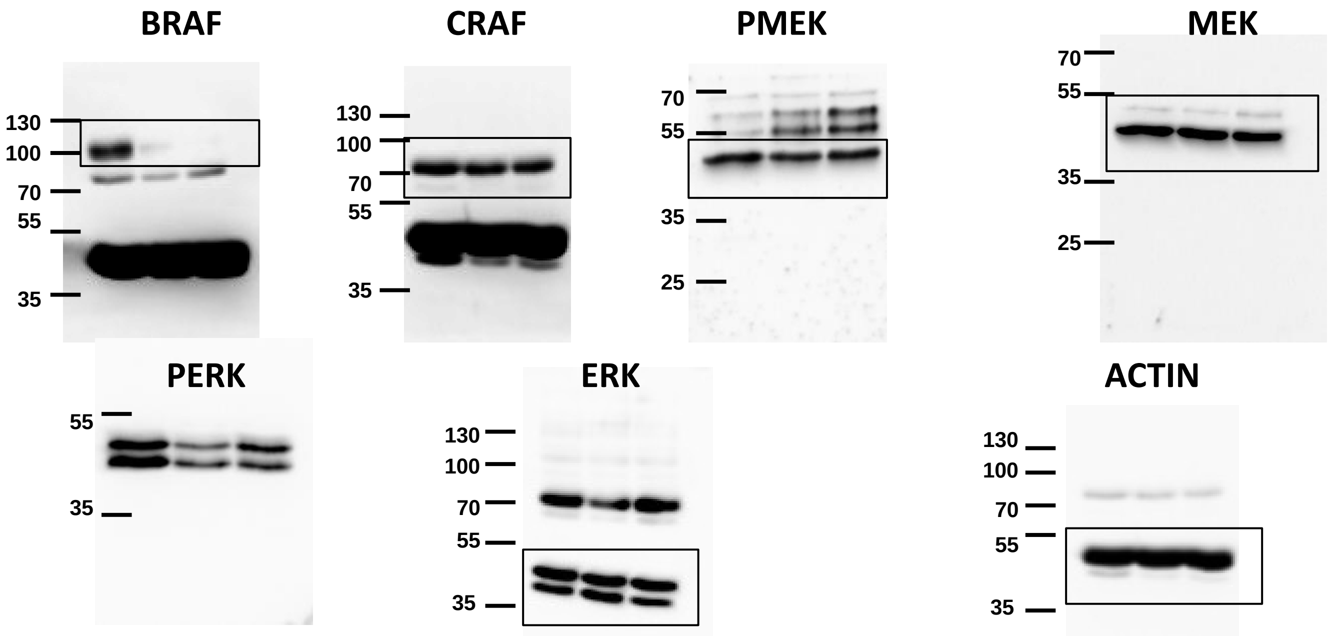

Figure 3f

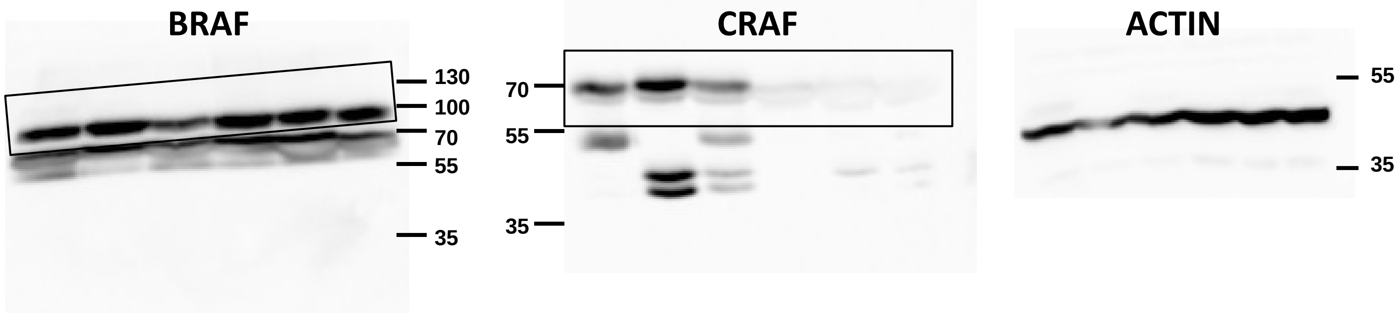

Figure 3h

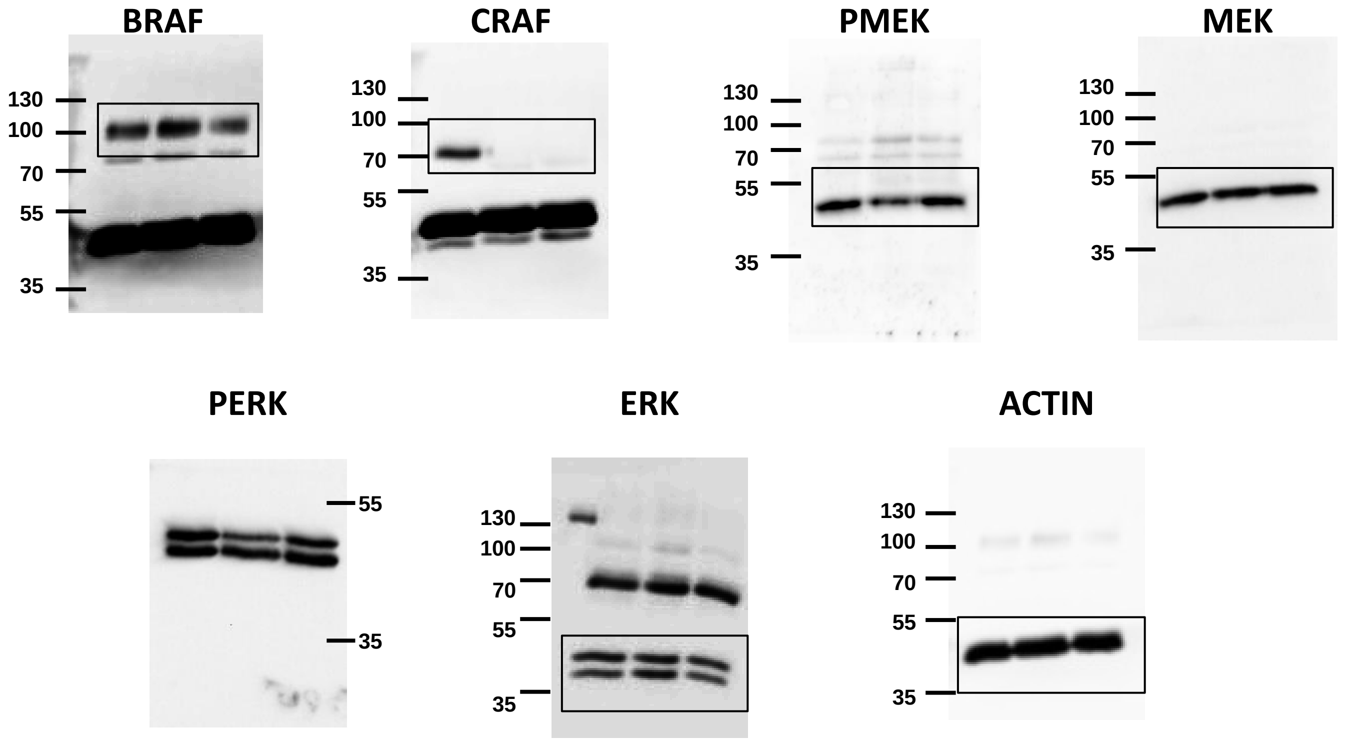

Figure 4c

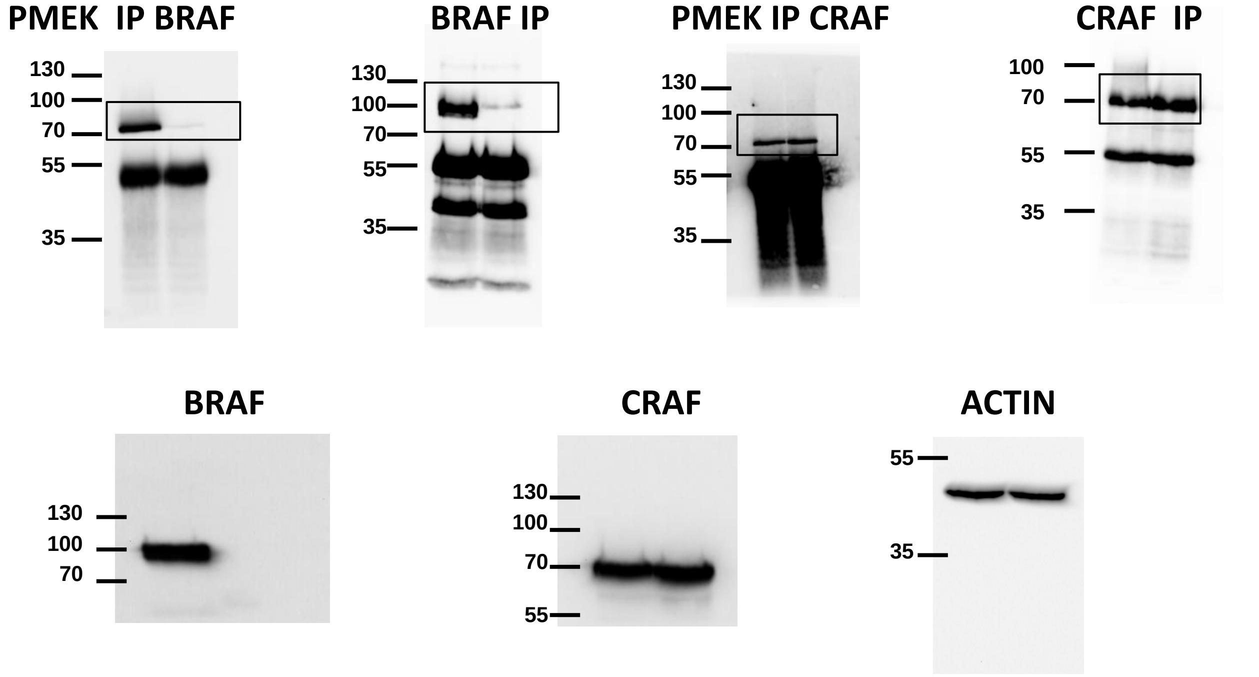

Figure 4d

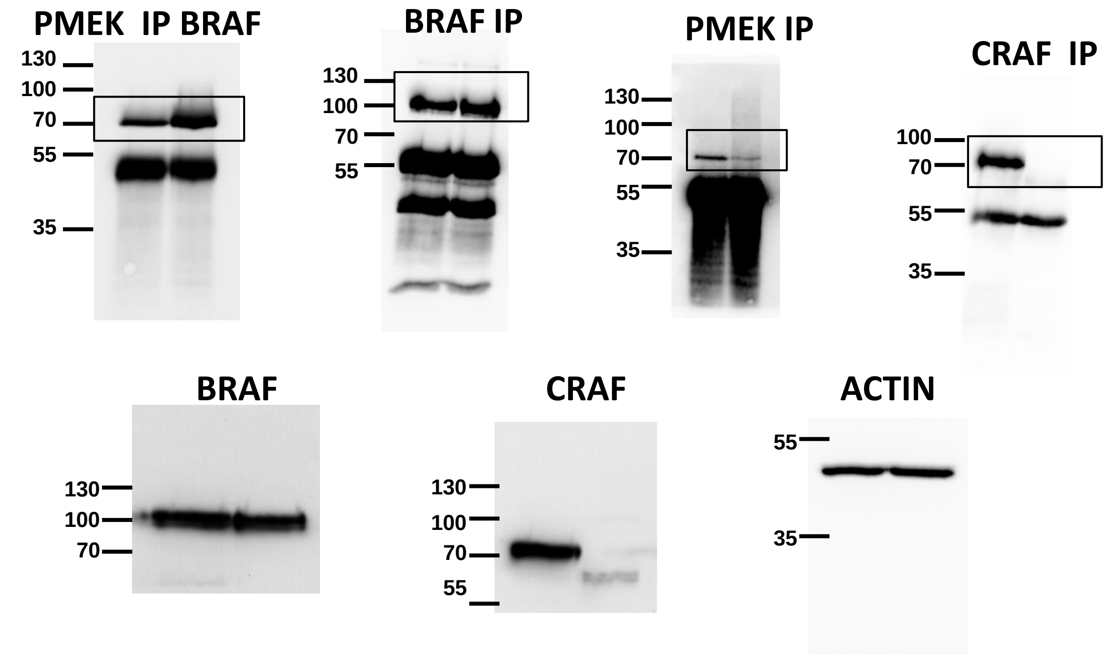

Figure 5a WM1361

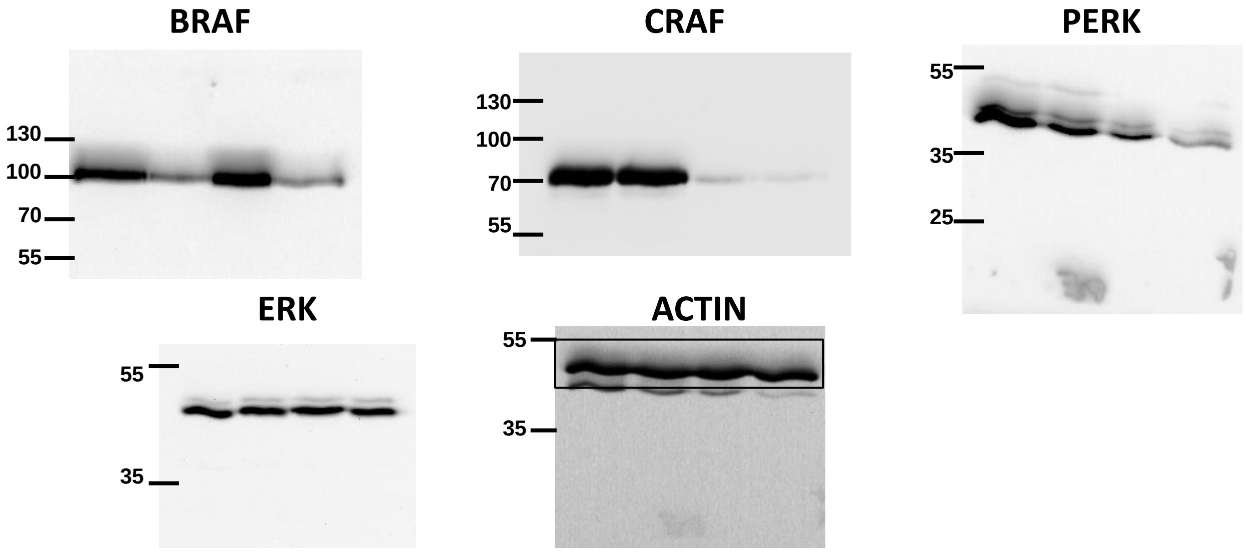

Figure 5a WM852

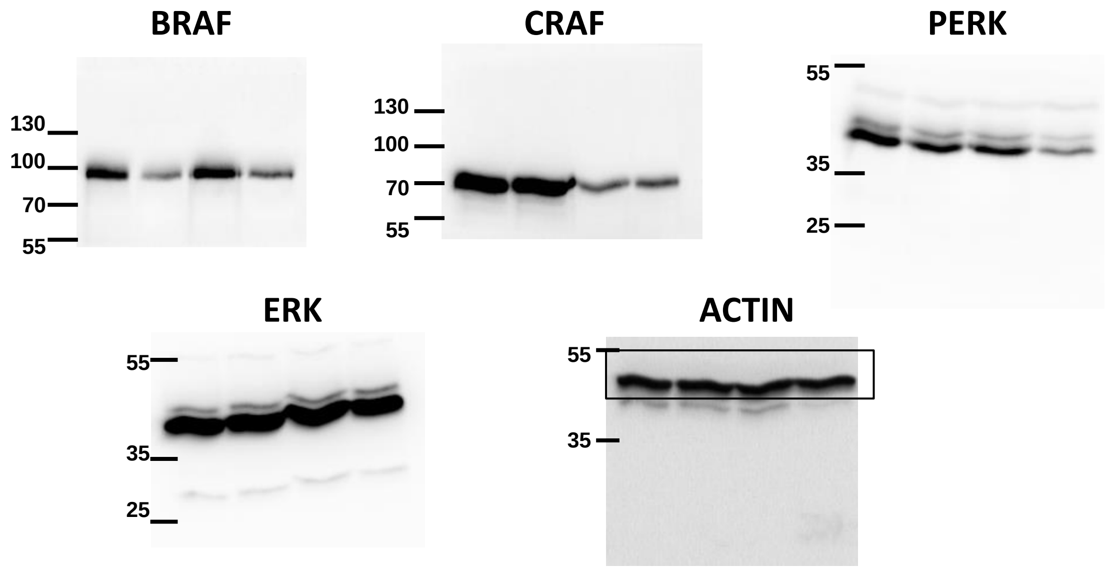

Figure 5a SbCl2

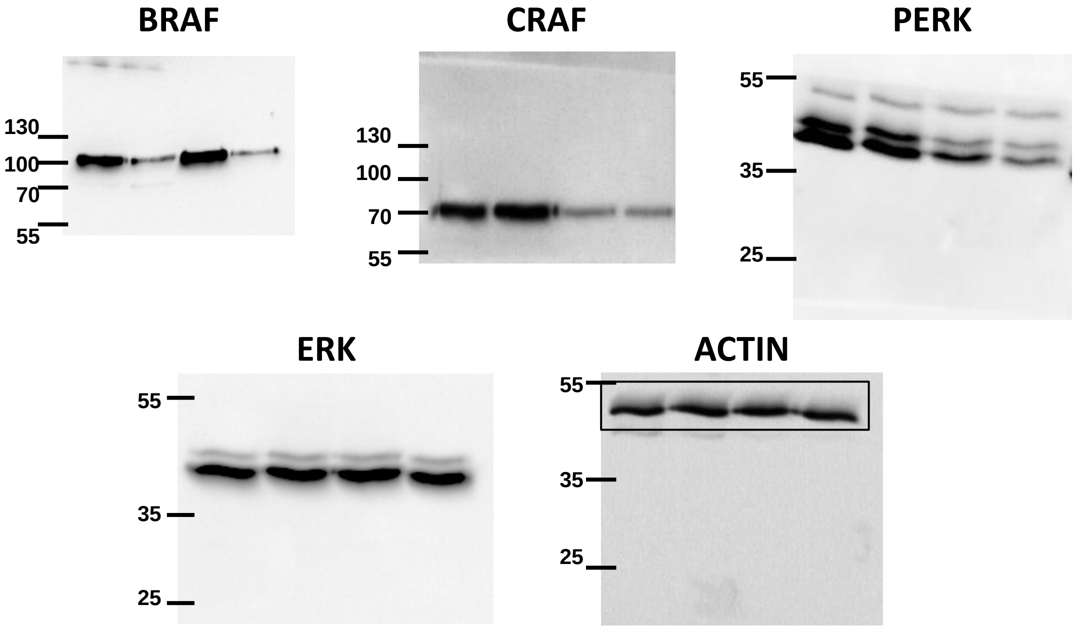

Figure 6b

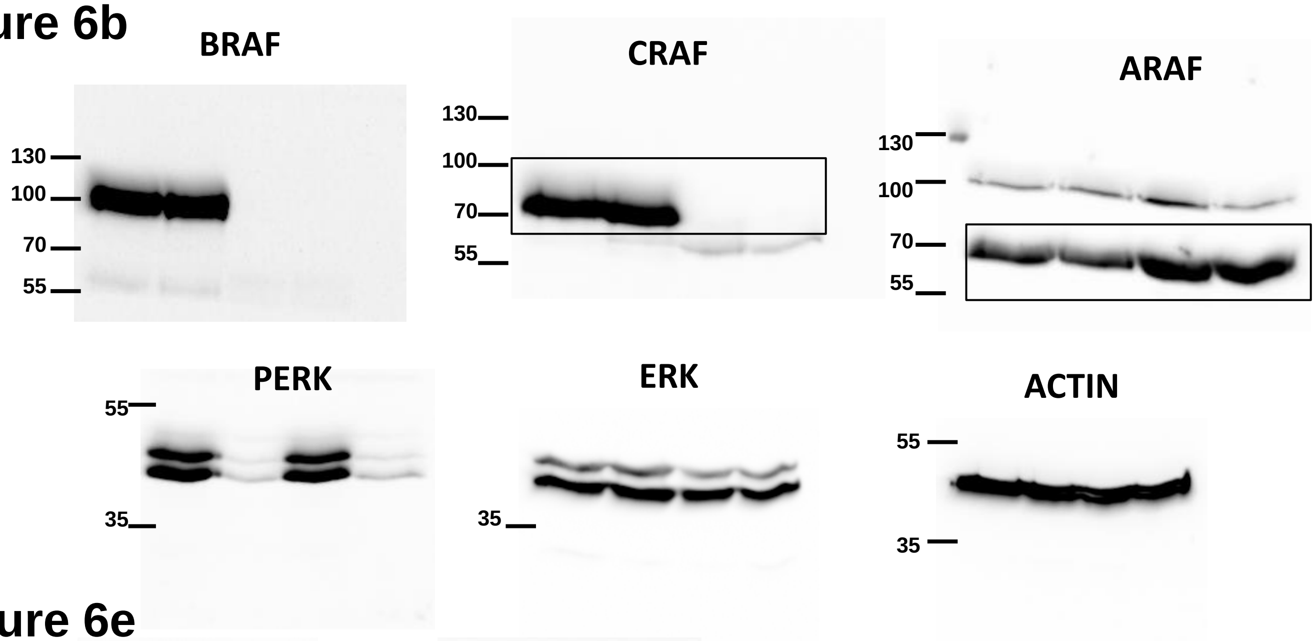

Figure 6e

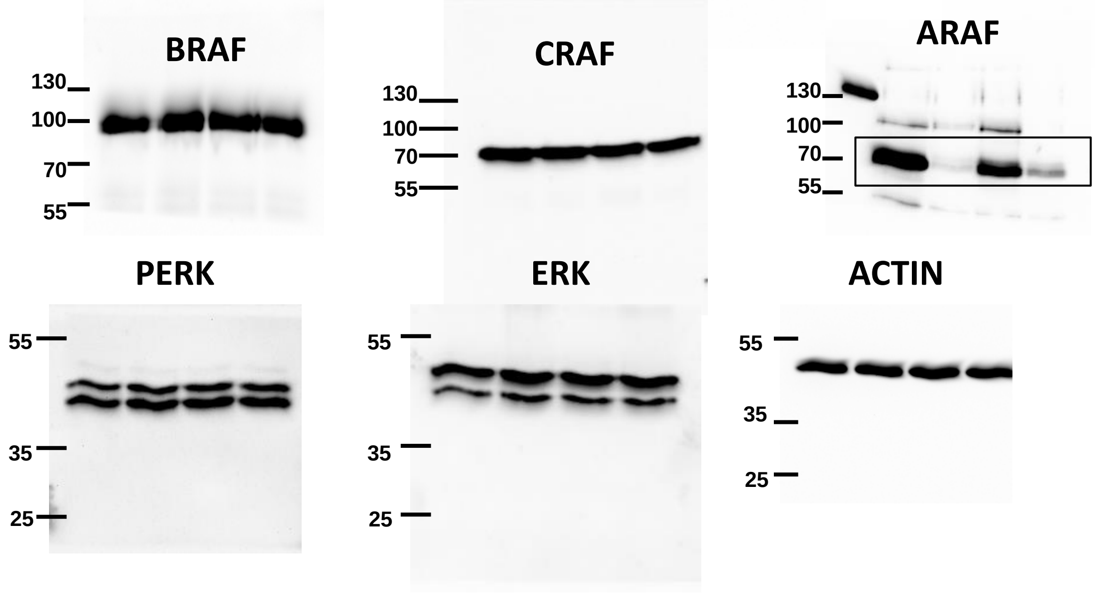

Figure 6g

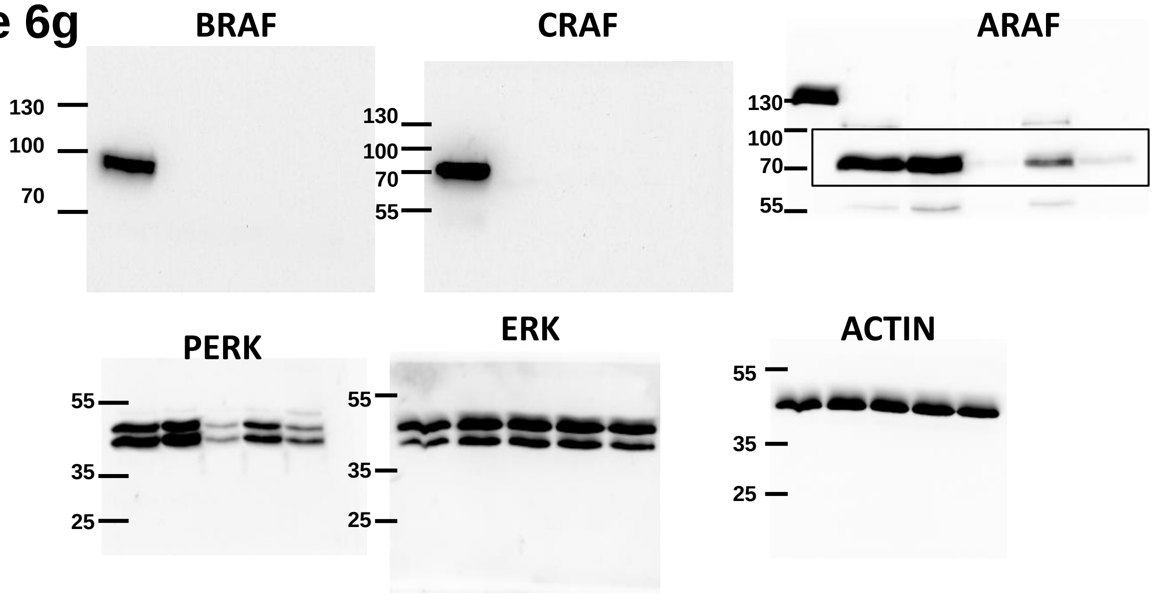

Figure 7a

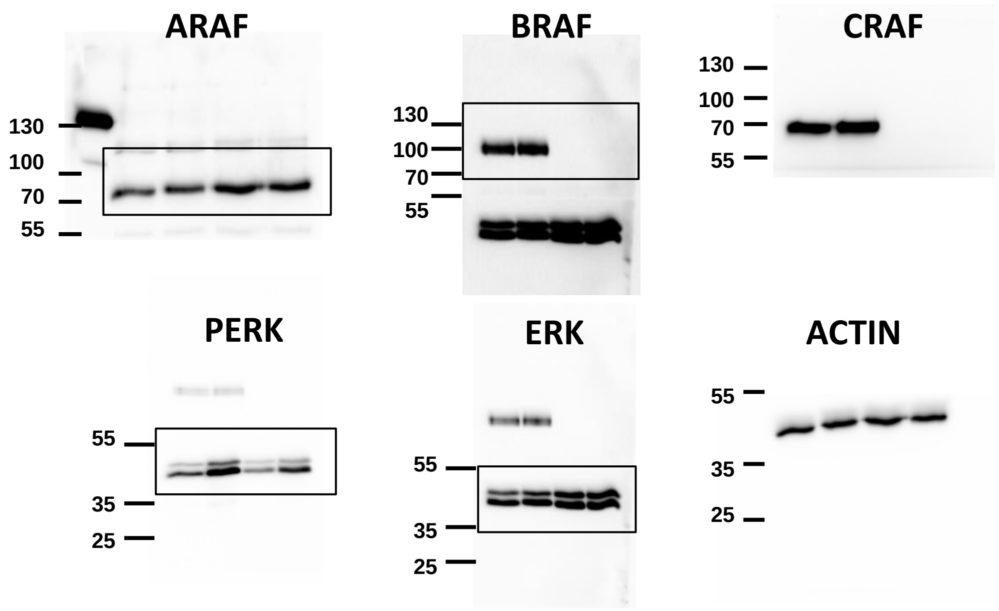

Figure 7b

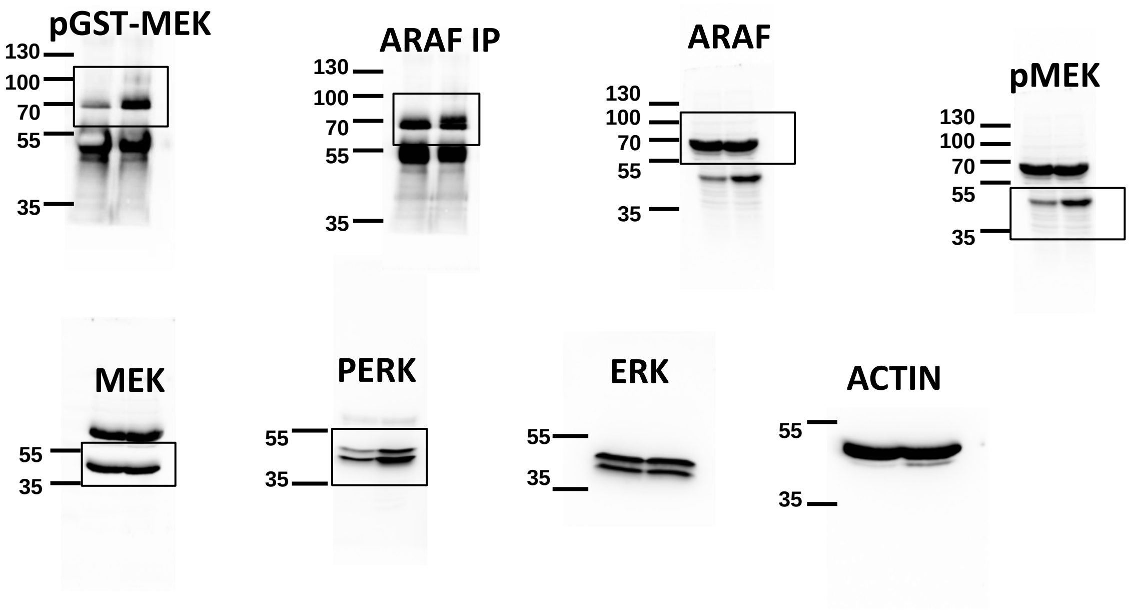

Supp Figure 5a

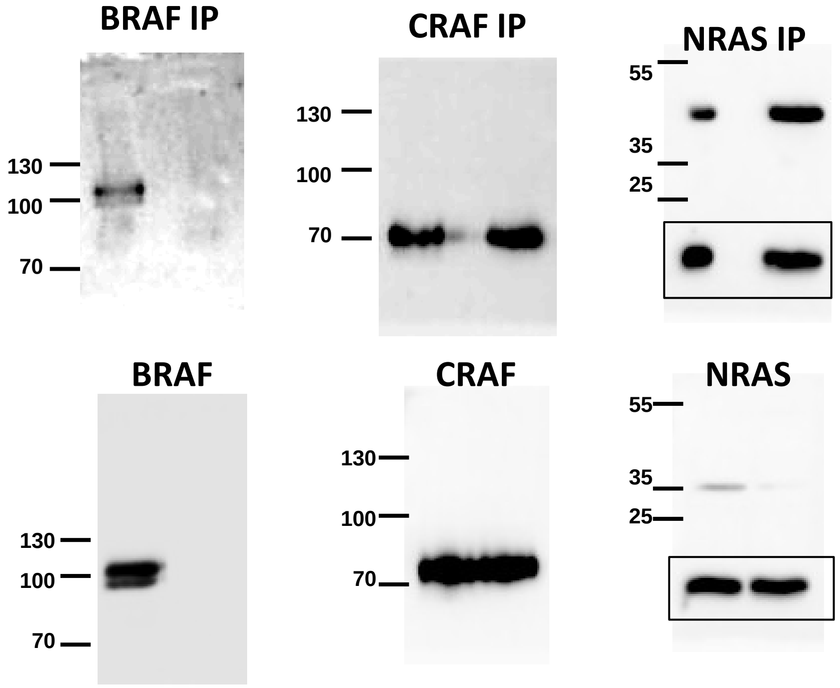

Supp Figure 5b

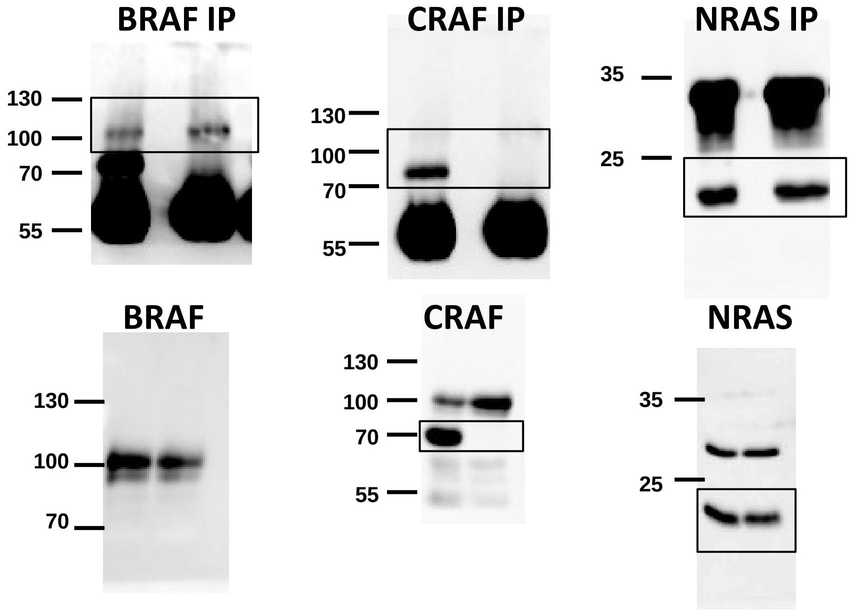

Supp Figure 7a WM1361

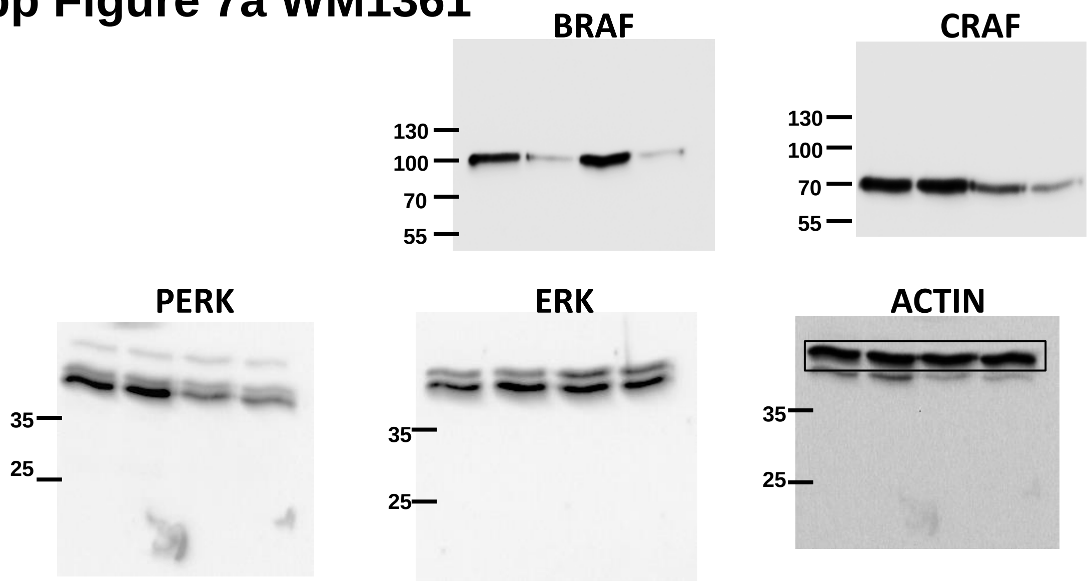

Supp Figure 7a WM852

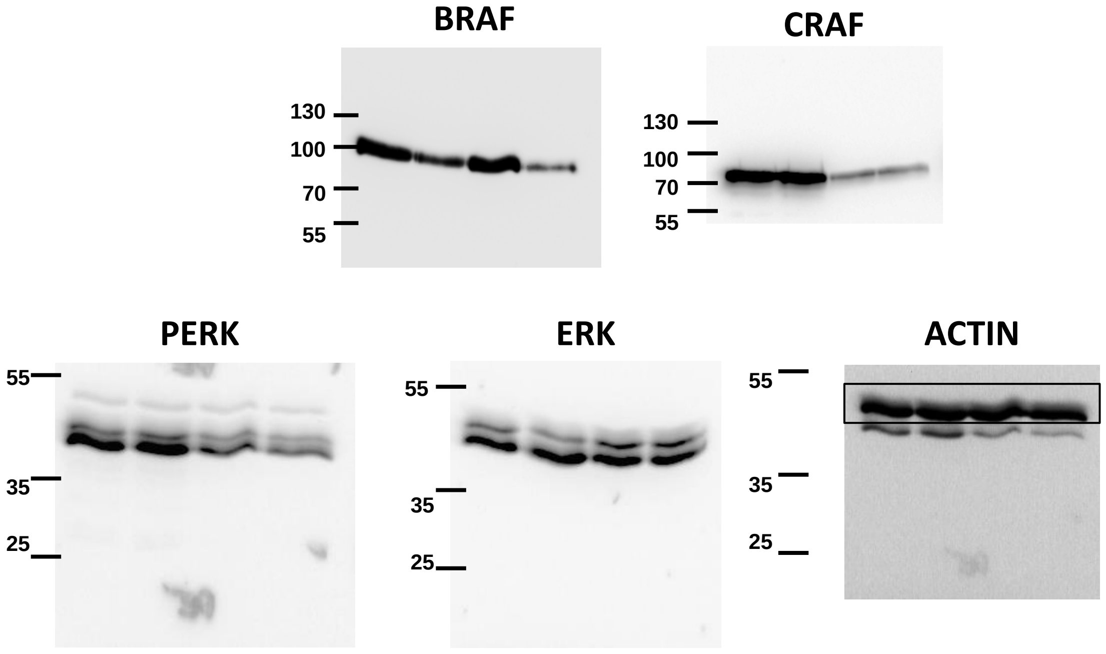

Supp Figure 7a SbCl2

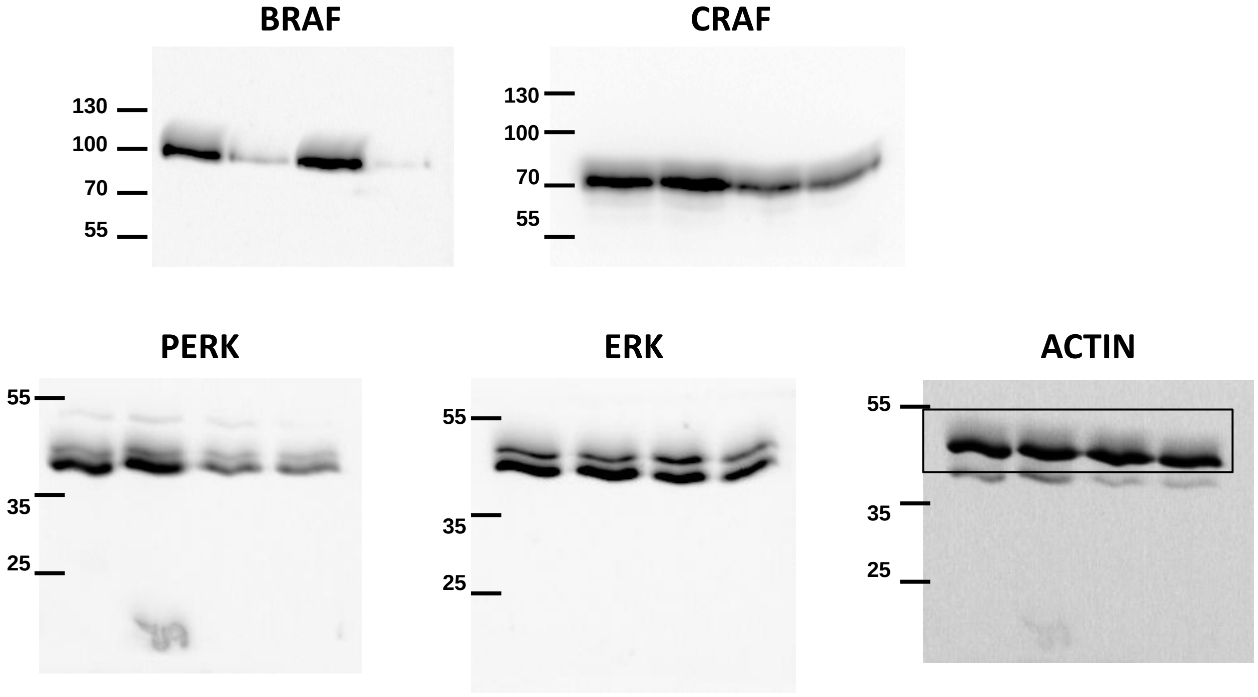

Supplement: Supplementary Information — Supplementary Figures [file ncomms15262-s1.pdf]
